# Supplementary figures and images for: Specific proteolysis mediated by a p97-directed proteolysis-targeting chimera (p97-PROTAC)
Source: eLife. 2025 Nov 26;14:e101496. doi: 10.7554/eLife.101496 (PMC12755880; doi:10.7554/eLife.101496)

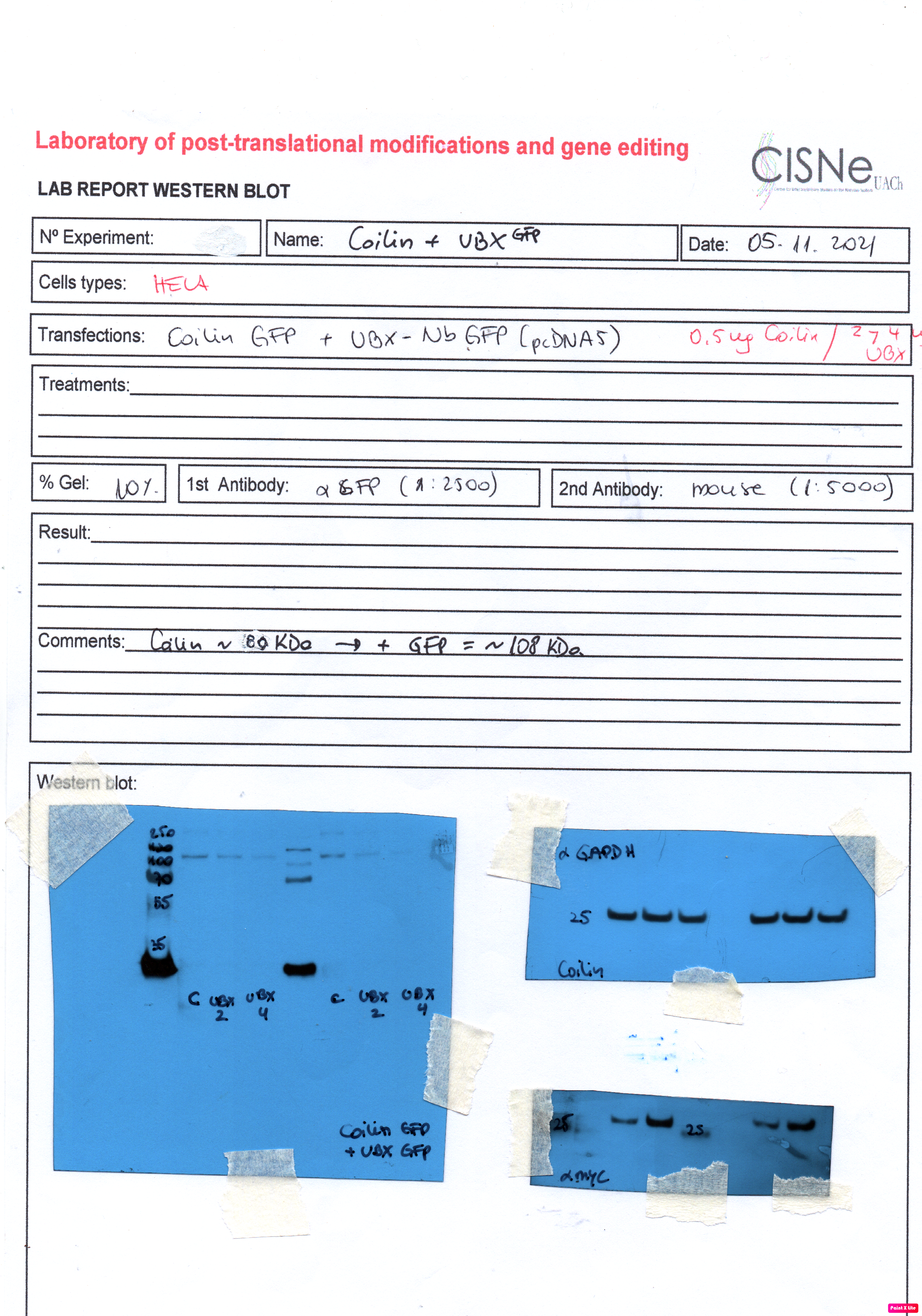

Supplement: Figure 1—source data 1. [file elife-101496-fig1-data1.zip › Figure 1-source data 1/Figure 1C-source data 1.tif]

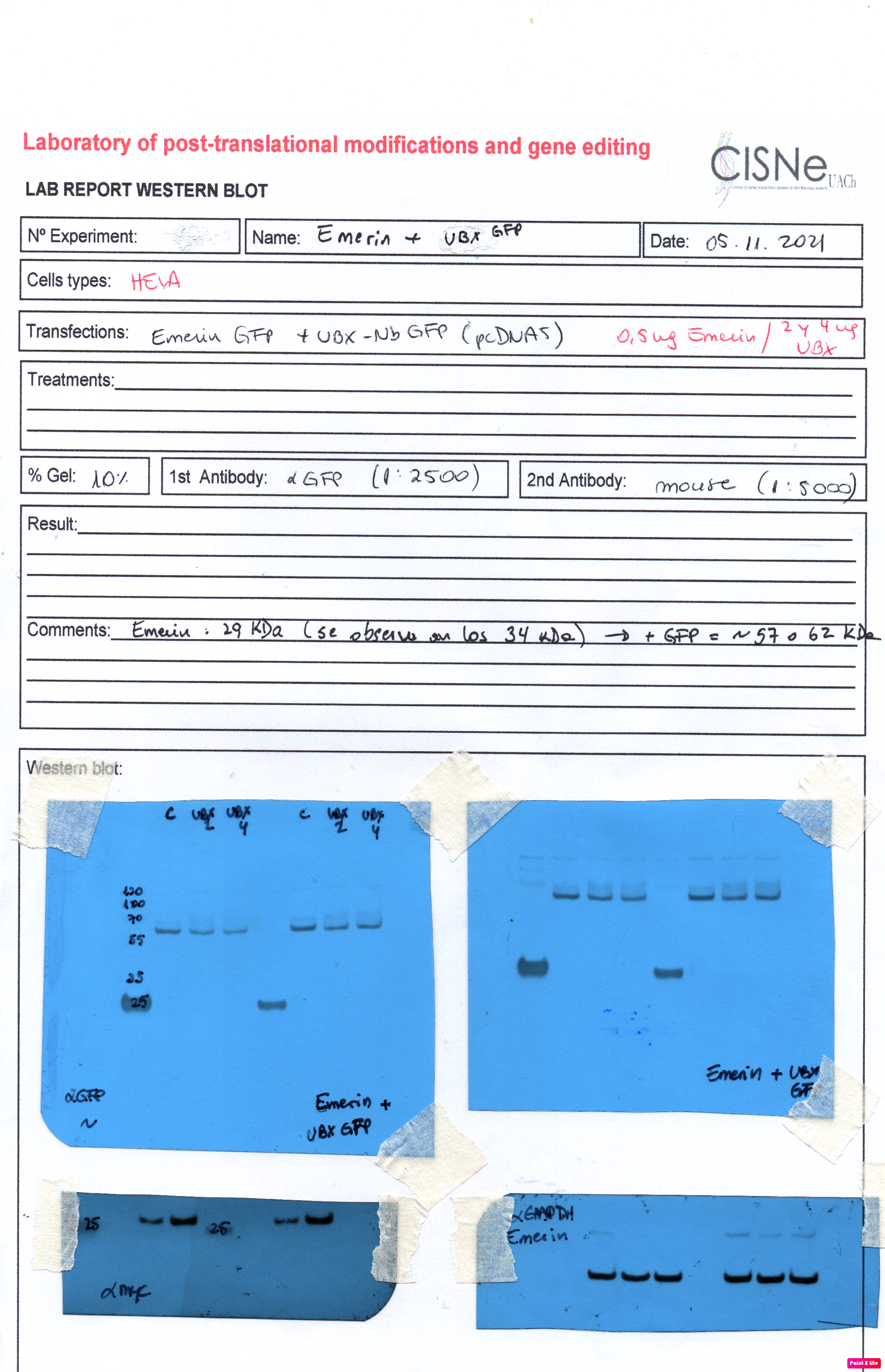

Supplement: Figure 1—source data 1. [file elife-101496-fig1-data1.zip › Figure 1-source data 1/Figure 1E-source data 1.tif]

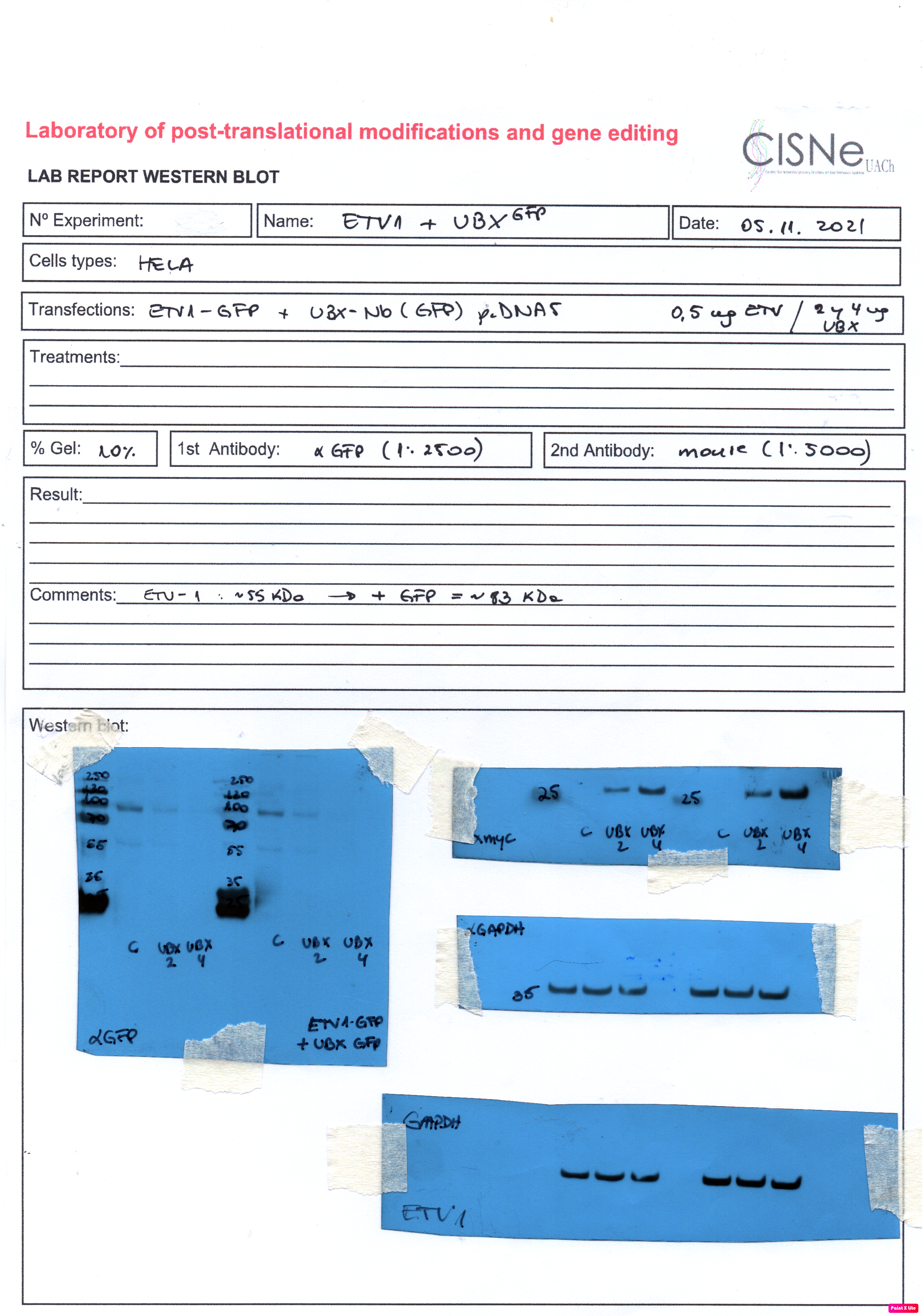

Supplement: Figure 1—source data 1. [file elife-101496-fig1-data1.zip › Figure 1-source data 1/Figure 1G-source data 1.tif]

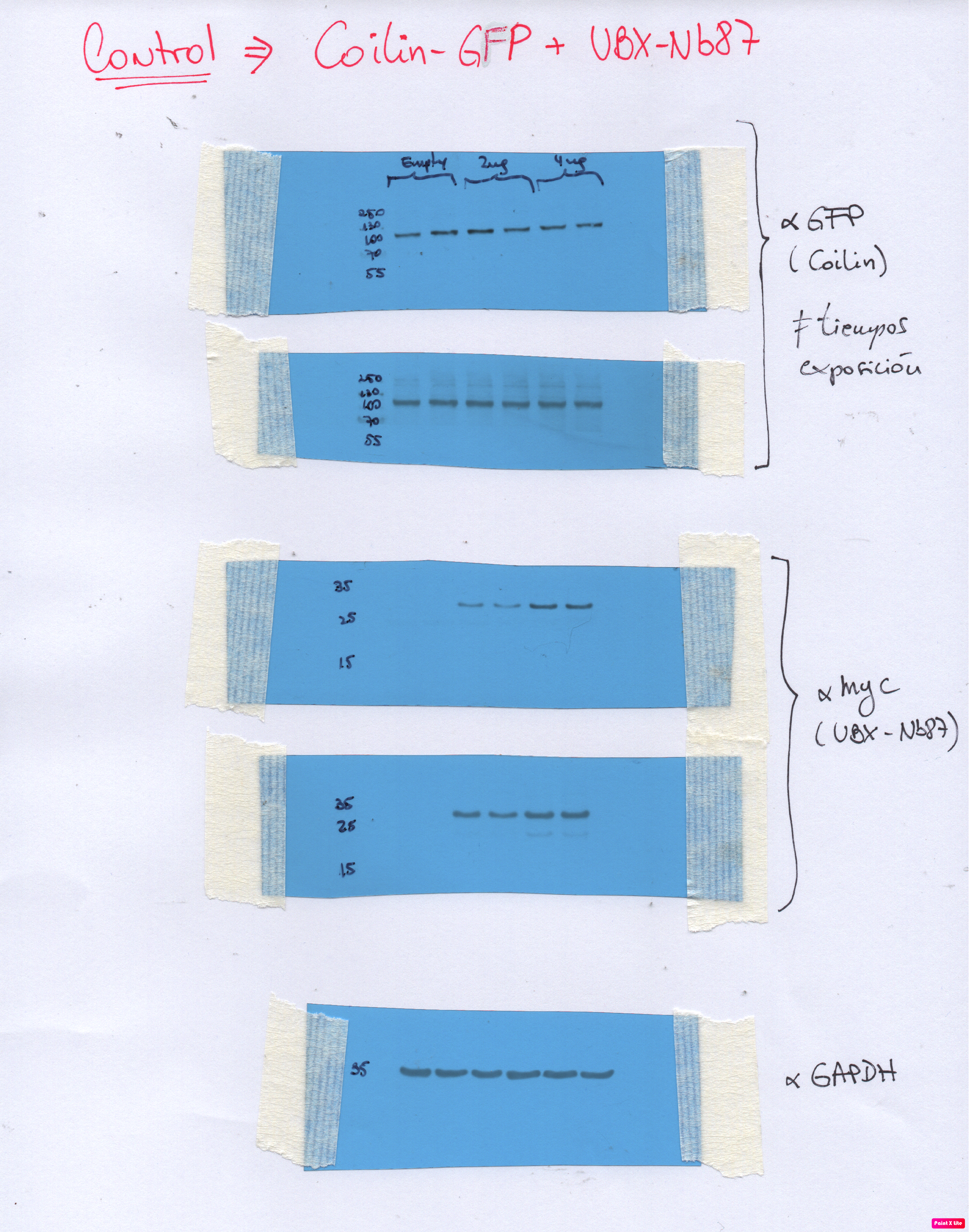

Supplement: Figure 1—figure supplement 1—source data 1. [file elife-101496-fig1-figsupp1-data1.zip › Figure 1-figure supplement 1-source data 1/Figure 1-figure supplement 1E-source data 1.tif]

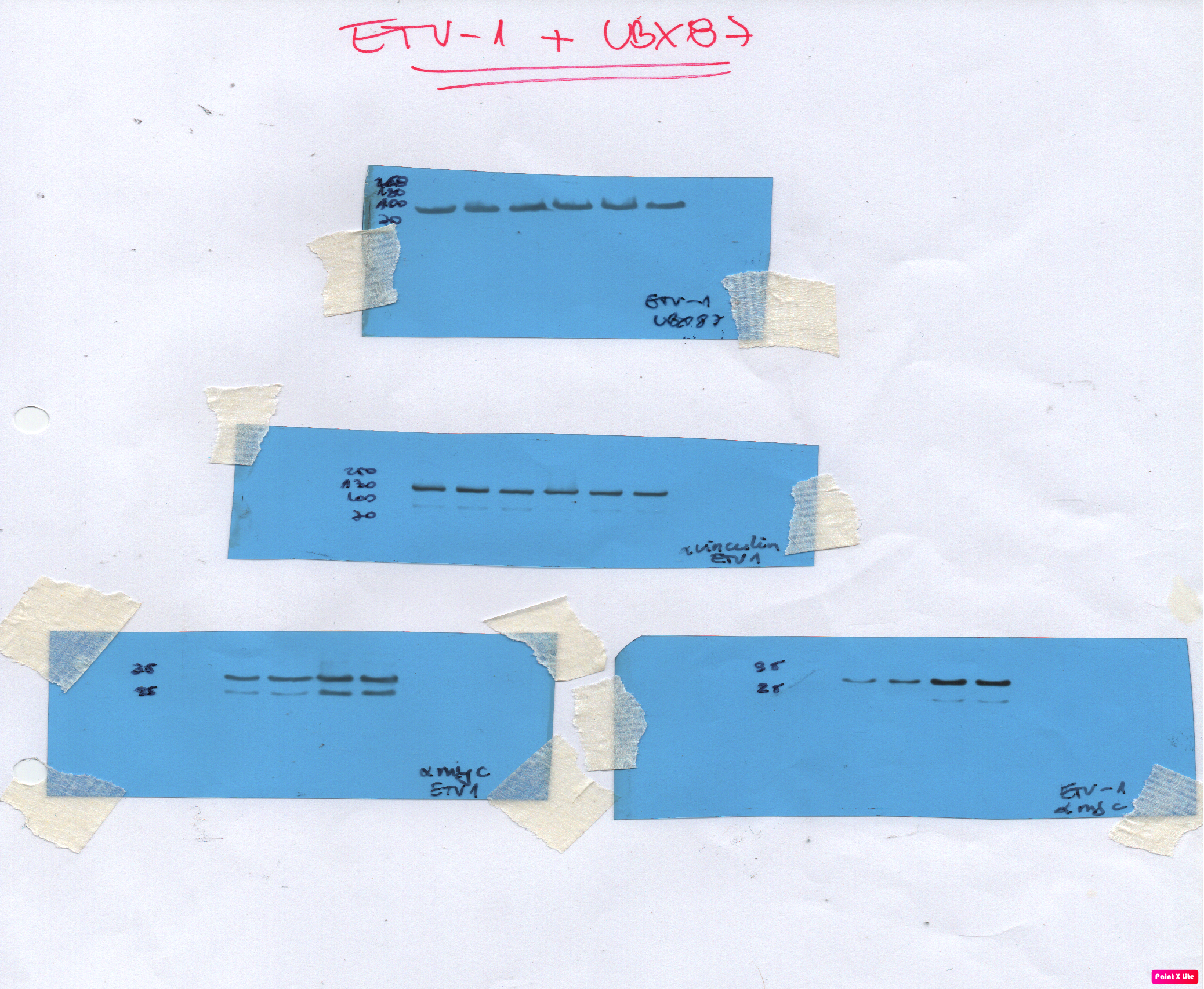

Supplement: Figure 1—figure supplement 1—source data 1. [file elife-101496-fig1-figsupp1-data1.zip › Figure 1-figure supplement 1-source data 1/Figure 1-figure supplement 1F-source data 1.tif]

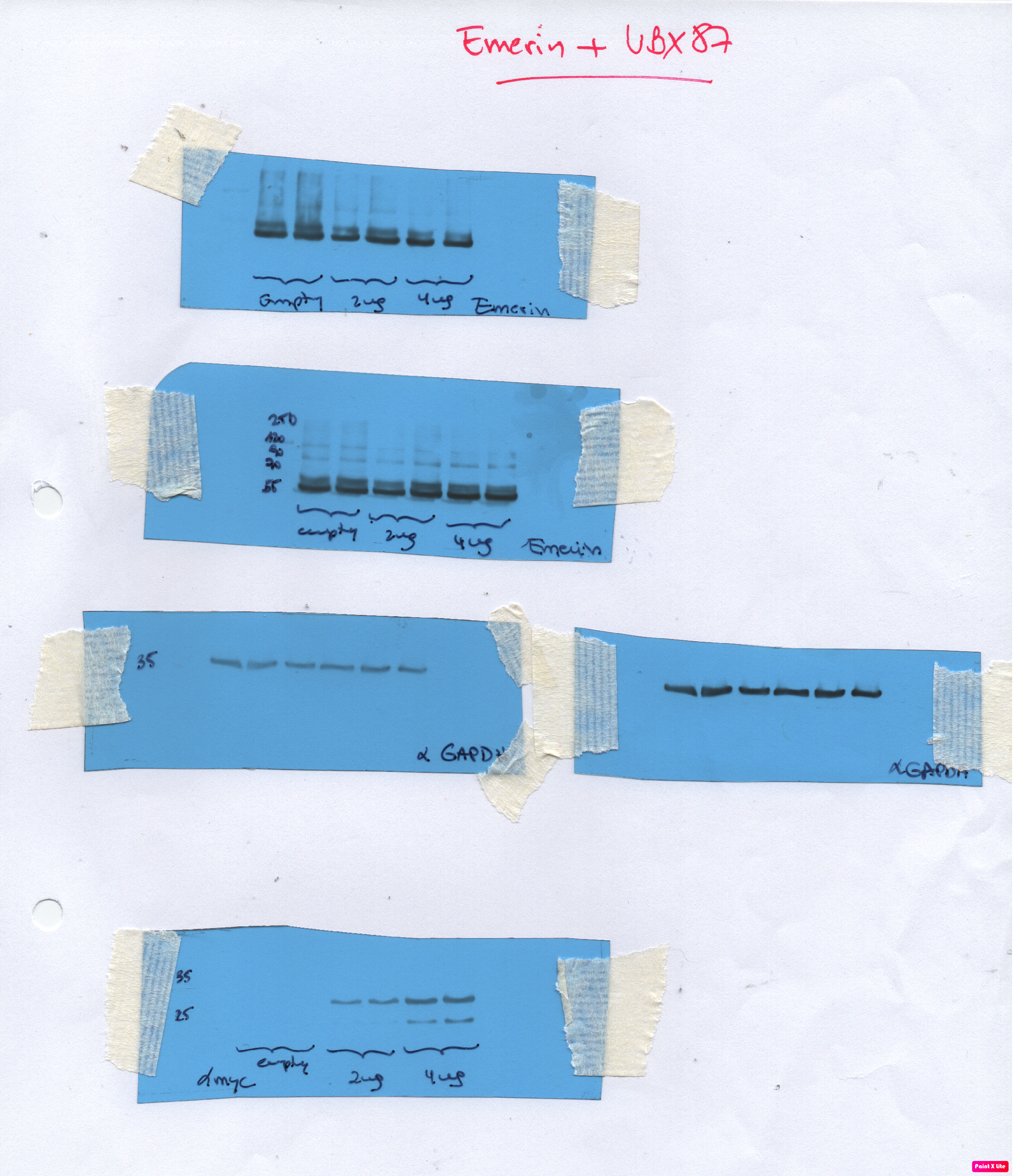

Supplement: Figure 1—figure supplement 1—source data 1. [file elife-101496-fig1-figsupp1-data1.zip › Figure 1-figure supplement 1-source data 1/Figure 1-figure supplement 1G-source data 1.tif]

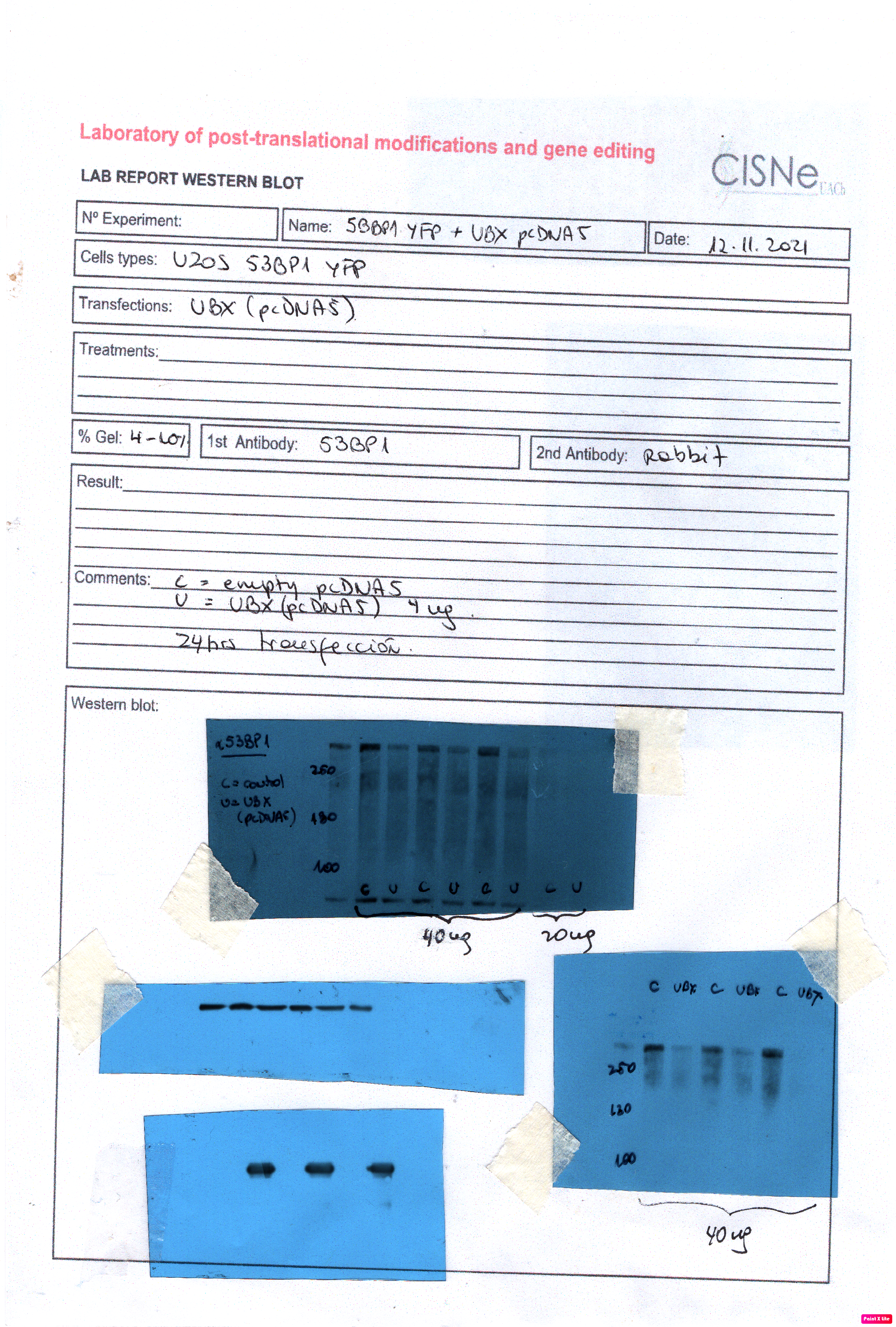

Supplement: Figure 2—source data 1. [file elife-101496-fig2-data1.zip › Figure 2-source data 1/Figure 2F-source data 1.tif]

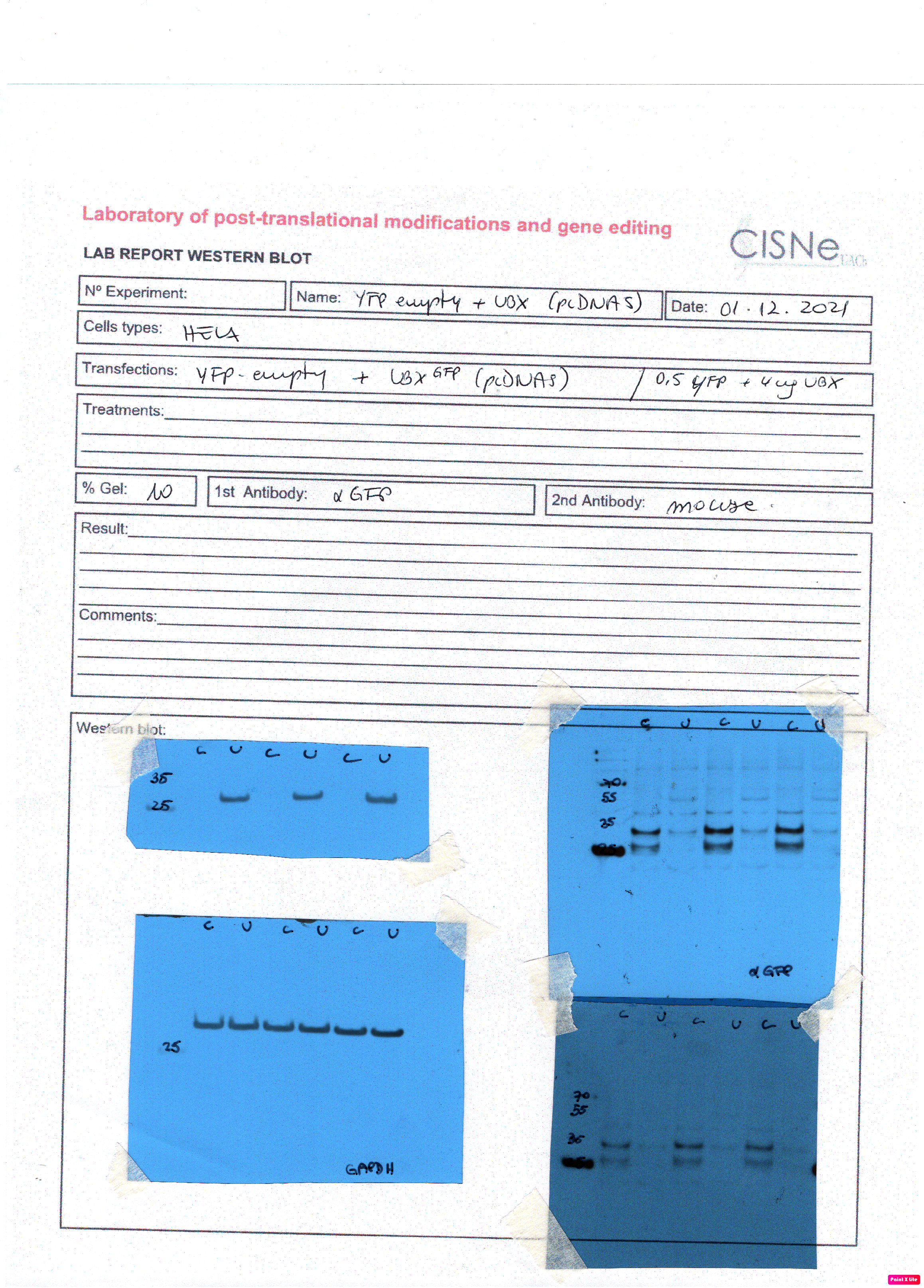

Supplement: Figure 4—source data 1. [file elife-101496-fig4-data1.zip › Figure 4-source data 1/Figure 4C-source data 1.tif]

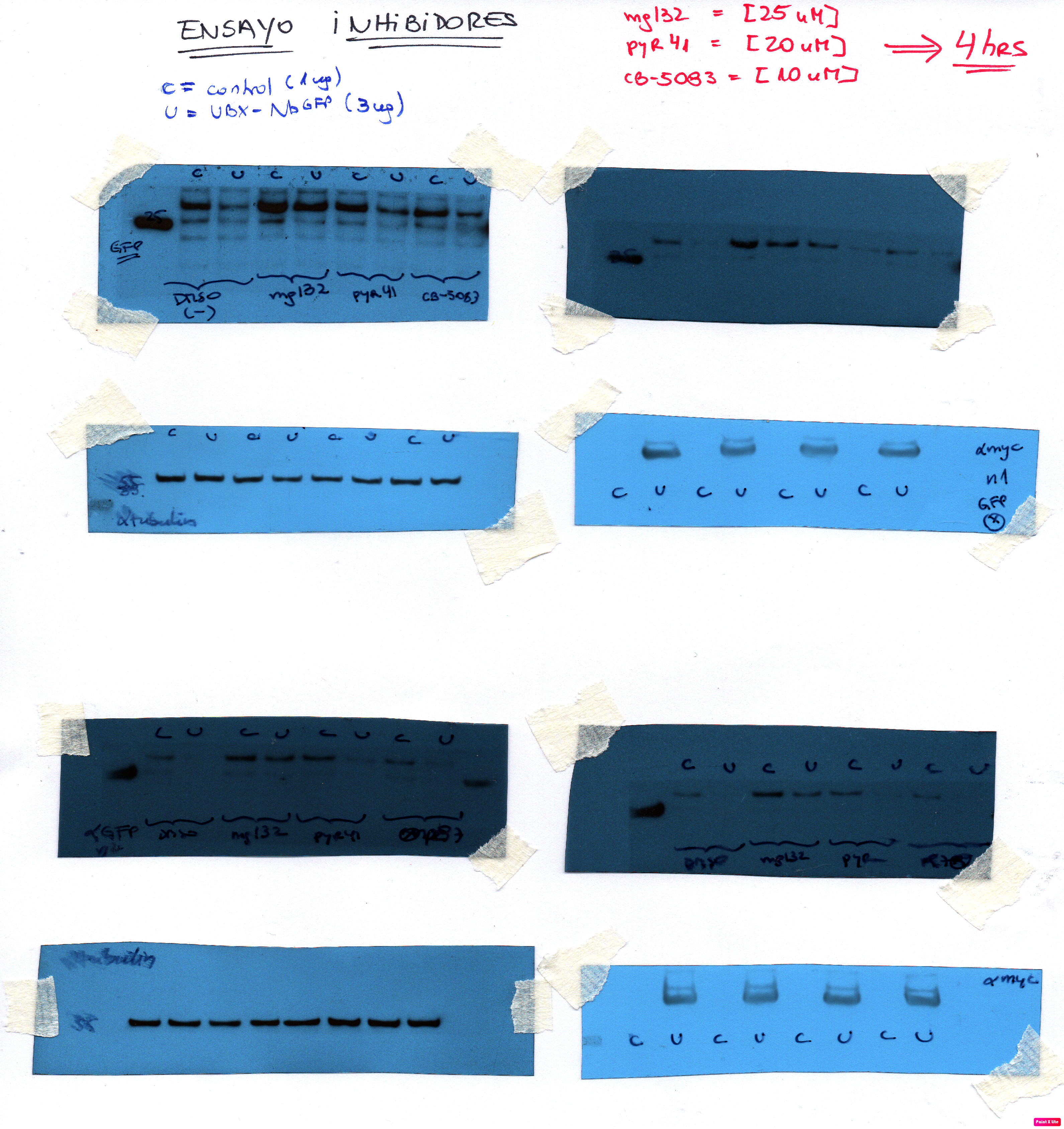

Supplement: Figure 4—source data 1. [file elife-101496-fig4-data1.zip › Figure 4-source data 1/Figure 4E-source data 1.tif]

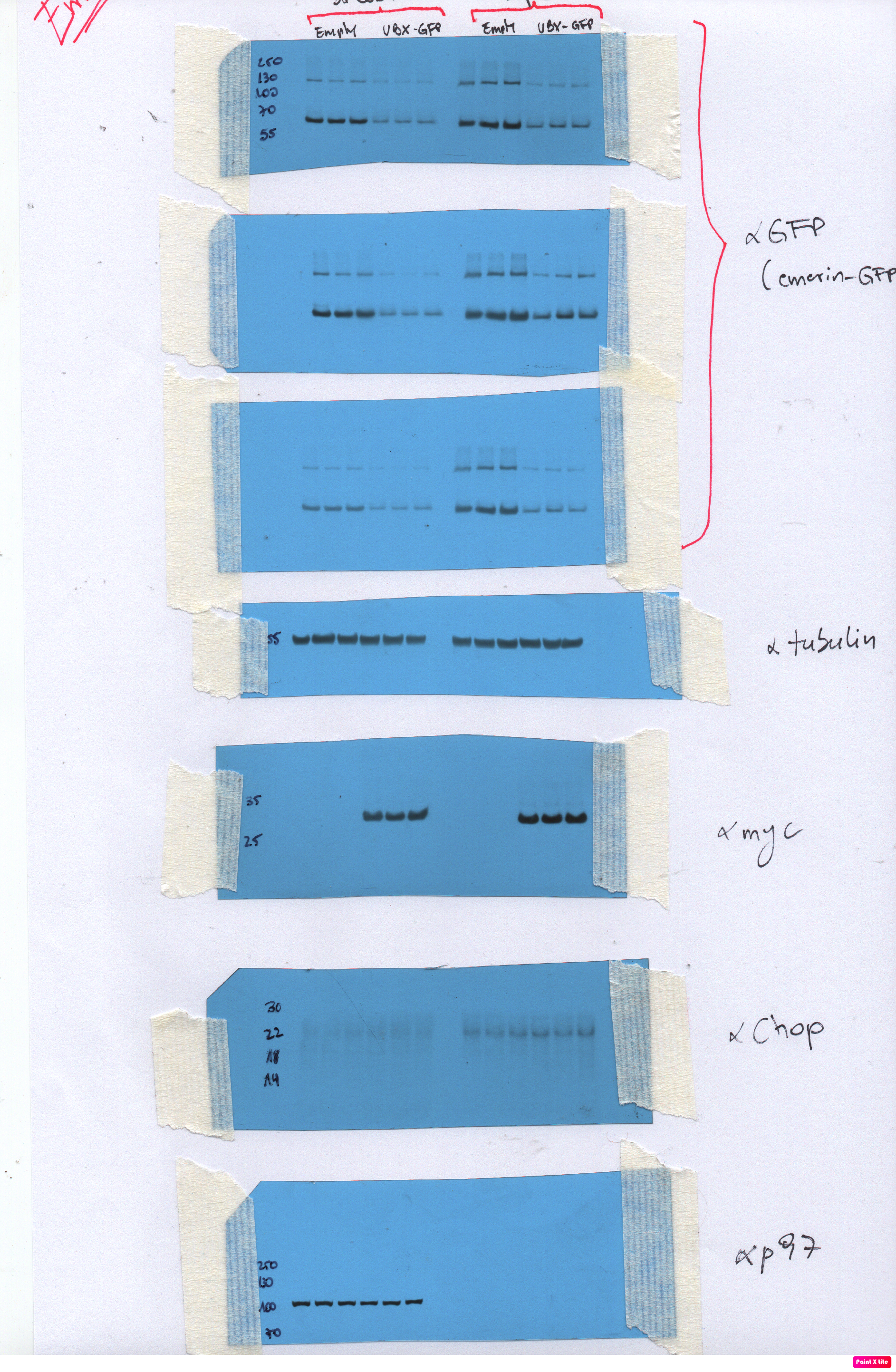

Supplement: Figure 4—source data 1. [file elife-101496-fig4-data1.zip › Figure 4-source data 1/Figure 4G-source data 1.tif]

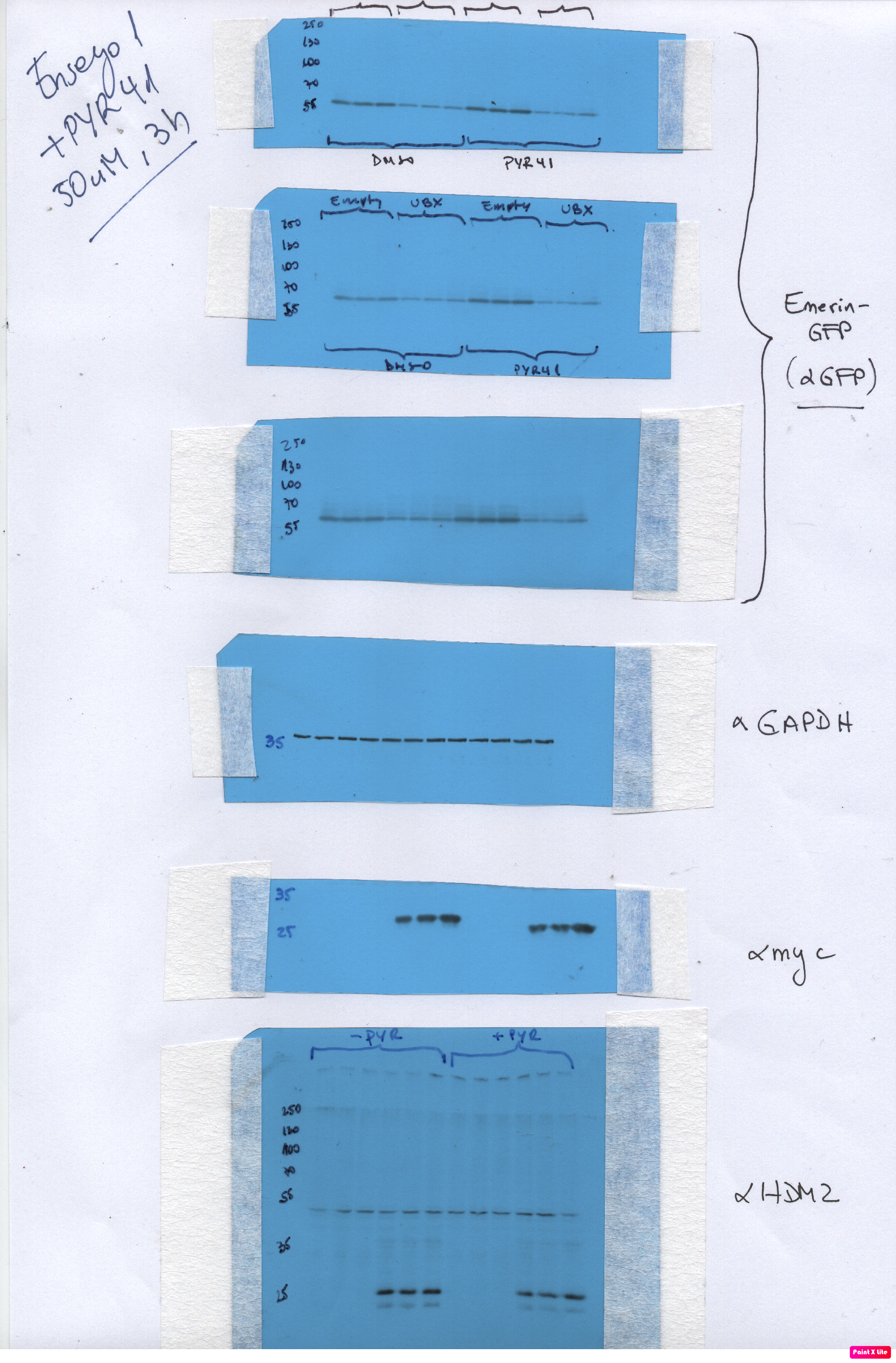

Supplement: Figure 4—source data 1. [file elife-101496-fig4-data1.zip › Figure 4-source data 1/Figure 4J_Assay1 Emerin PYR41-source data 1.tif]

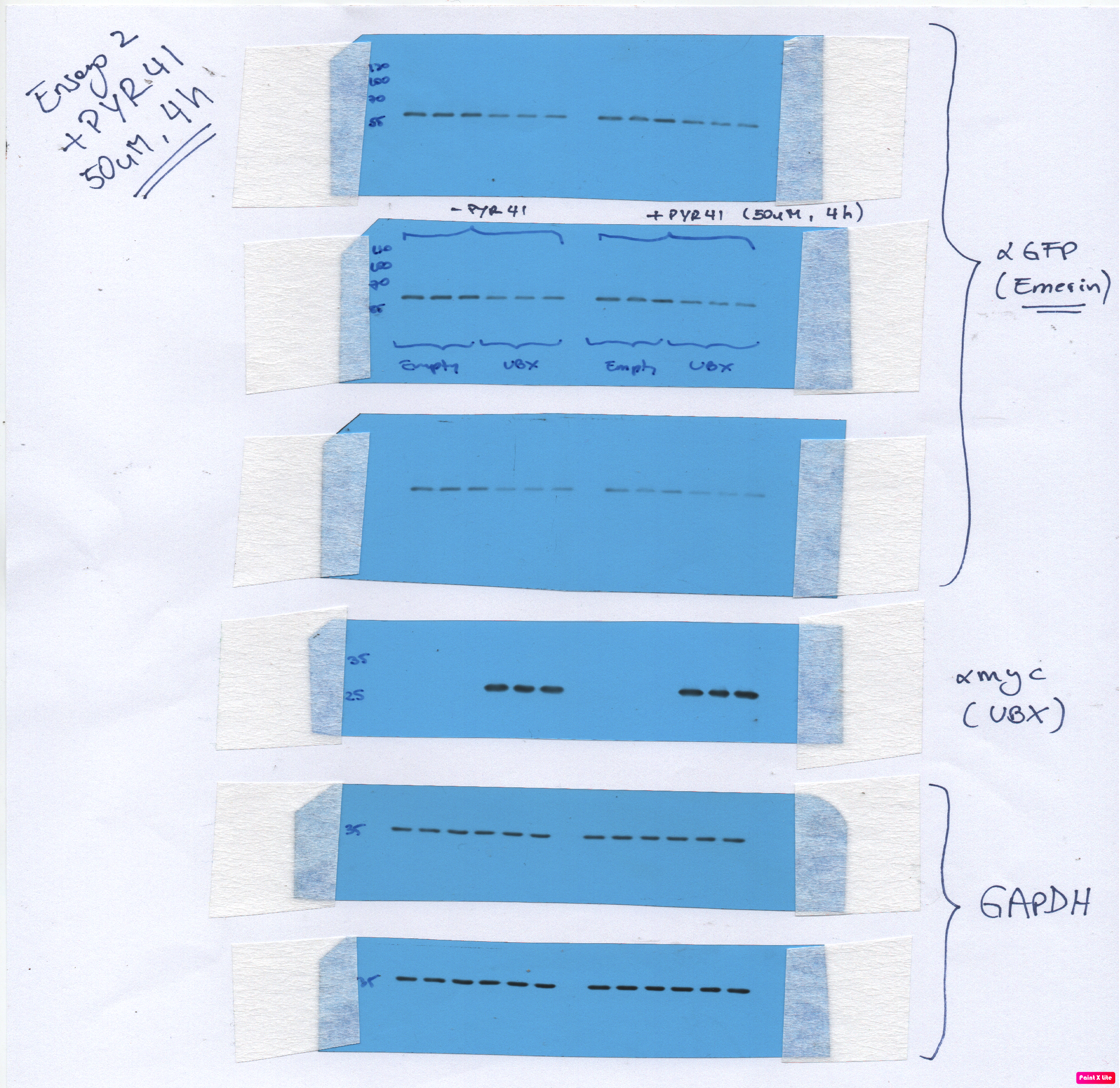

Supplement: Figure 4—source data 1. [file elife-101496-fig4-data1.zip › Figure 4-source data 1/Figure 4J_Assay2 Emerin PYR41-source data 1.tif]

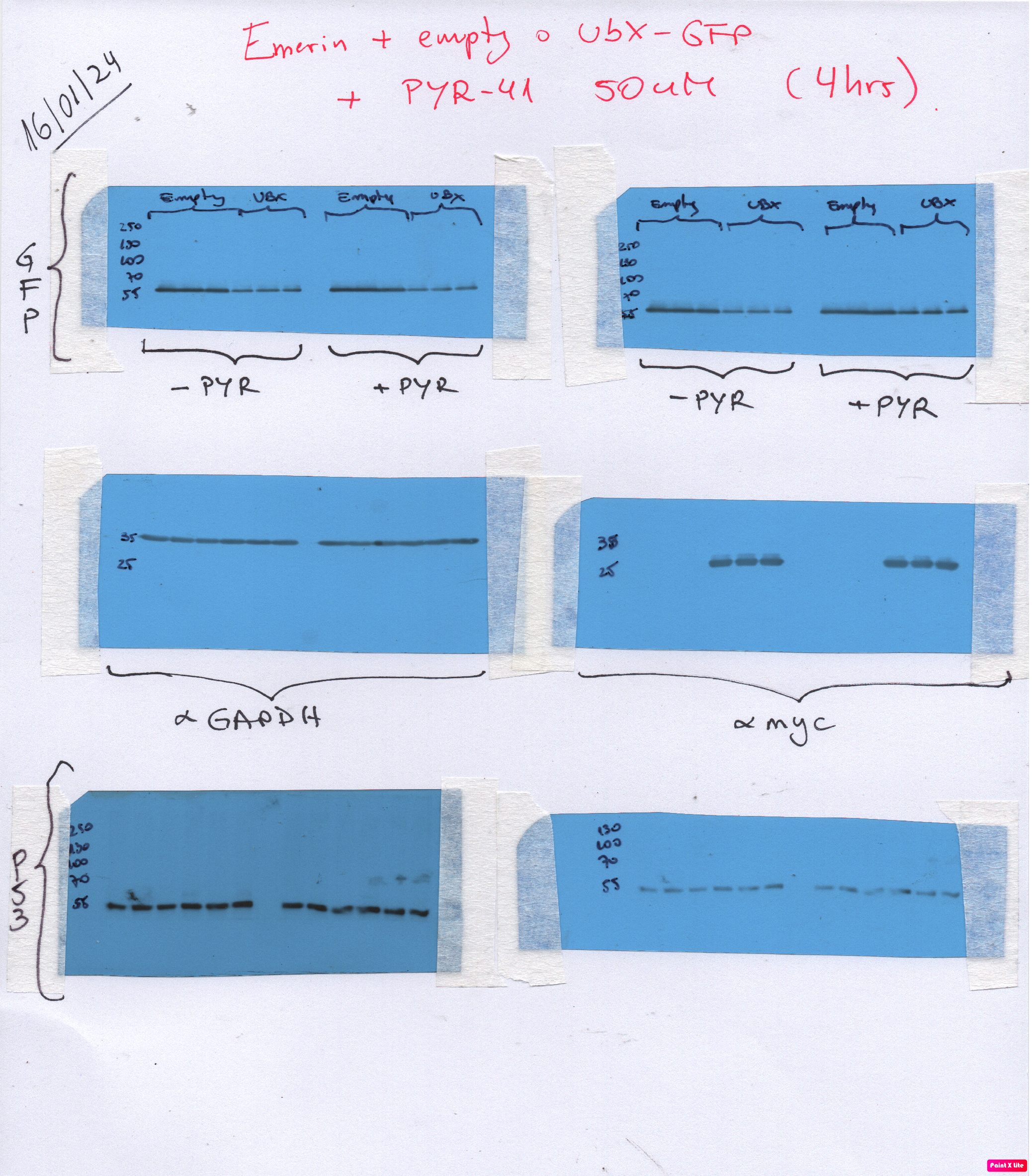

Supplement: Figure 4—source data 1. [file elife-101496-fig4-data1.zip › Figure 4-source data 1/Figure 4J_Assay3 Emerin PYR41-source data 1.tif]

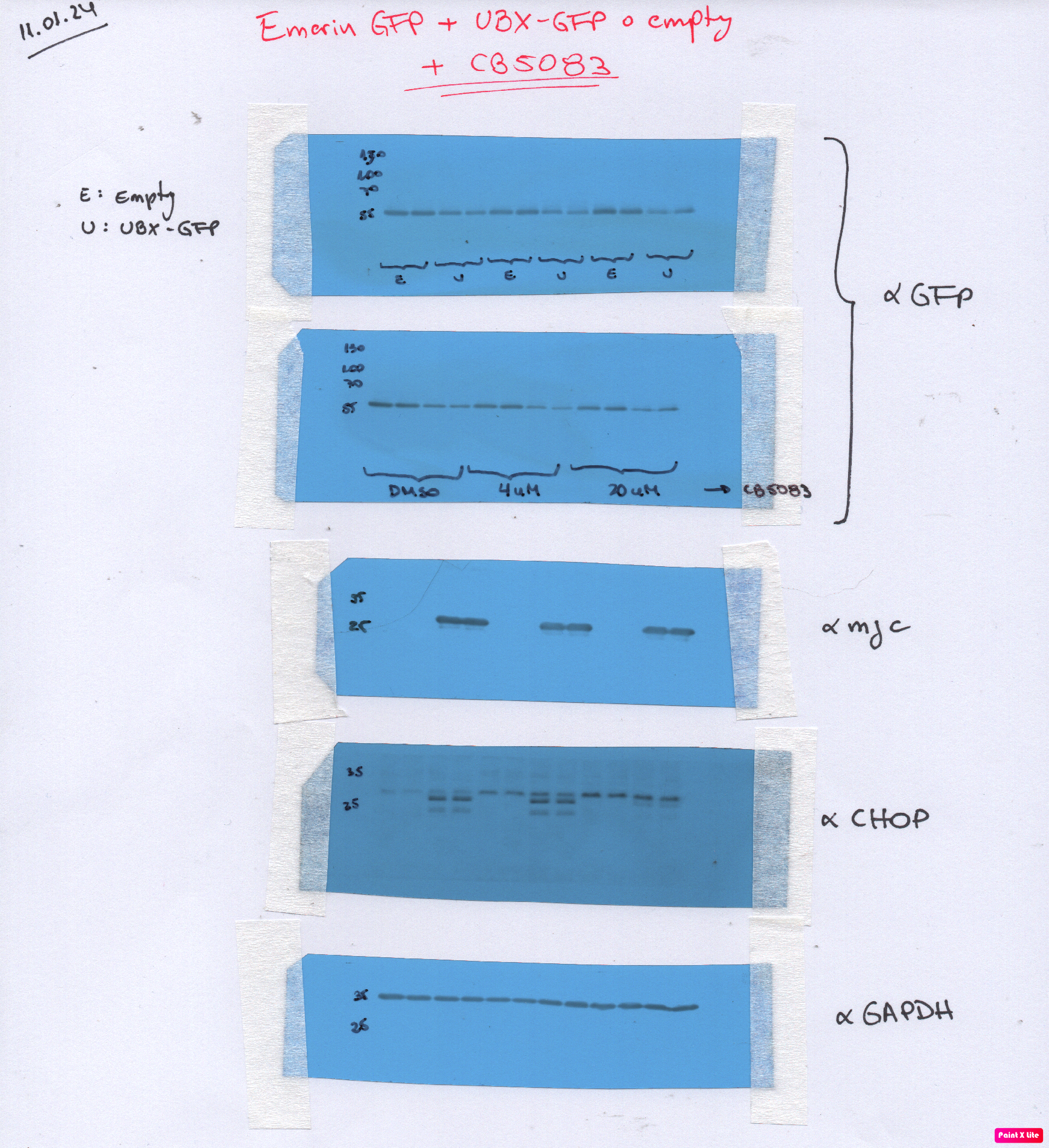

Supplement: Figure 4—source data 1. [file elife-101496-fig4-data1.zip › Figure 4-source data 1/Figure 4L-source data 1.tif]

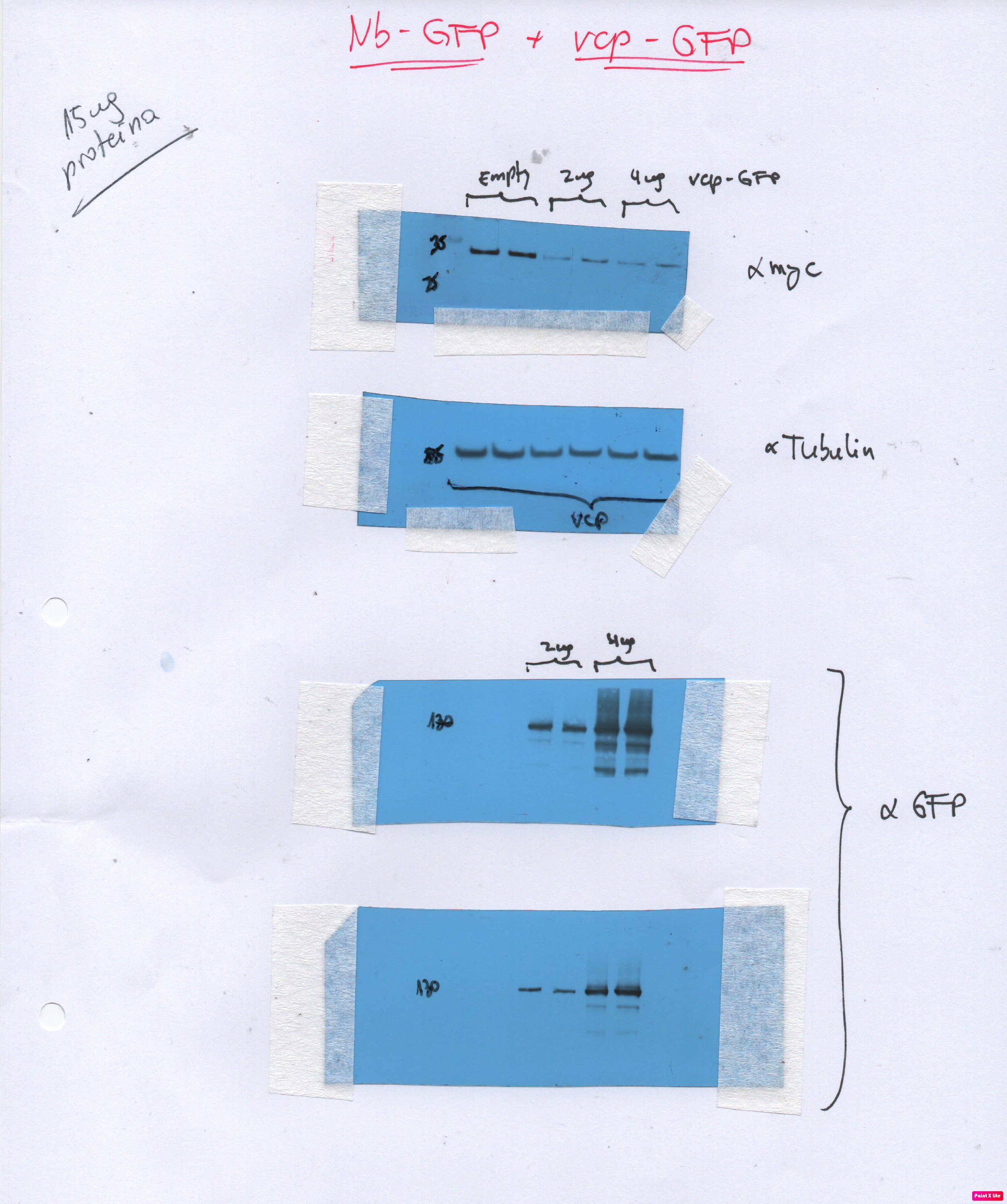

Supplement: Figure 4—figure supplement 1—source data 1. [file elife-101496-fig4-figsupp1-data1.zip › Figure 4-figure supplement 1-source data 1/Figure 4-figure supplement 1A-source data 1.tif]

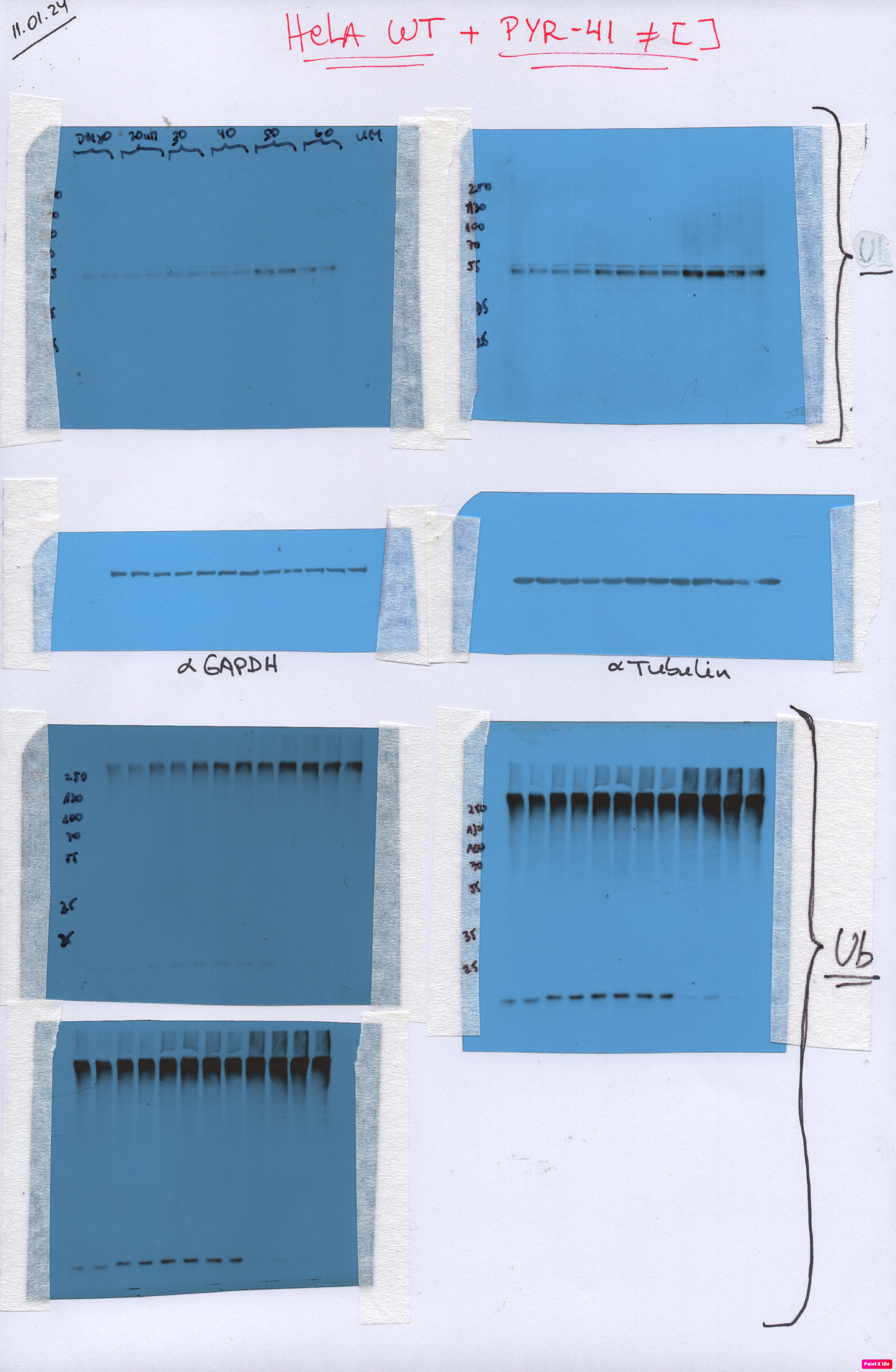

Supplement: Figure 4—figure supplement 1—source data 1. [file elife-101496-fig4-figsupp1-data1.zip › Figure 4-figure supplement 1-source data 1/Figure 4-figure supplement 1C-source data 1.tif]

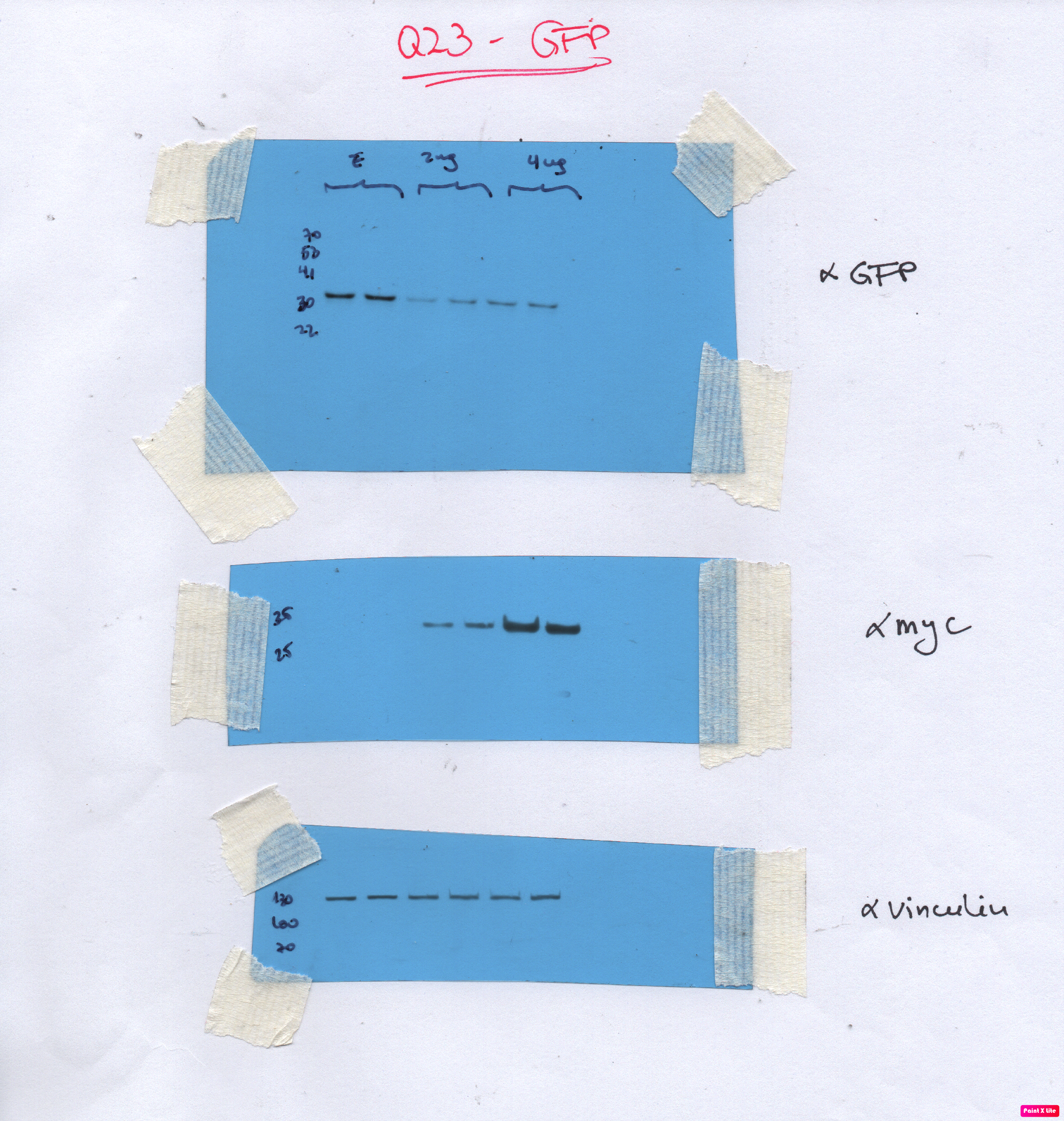

Supplement: Figure 5—source data 1. [file elife-101496-fig5-data1.zip › Figure 5-source data 1/Figure 5A-source data 1.tif]

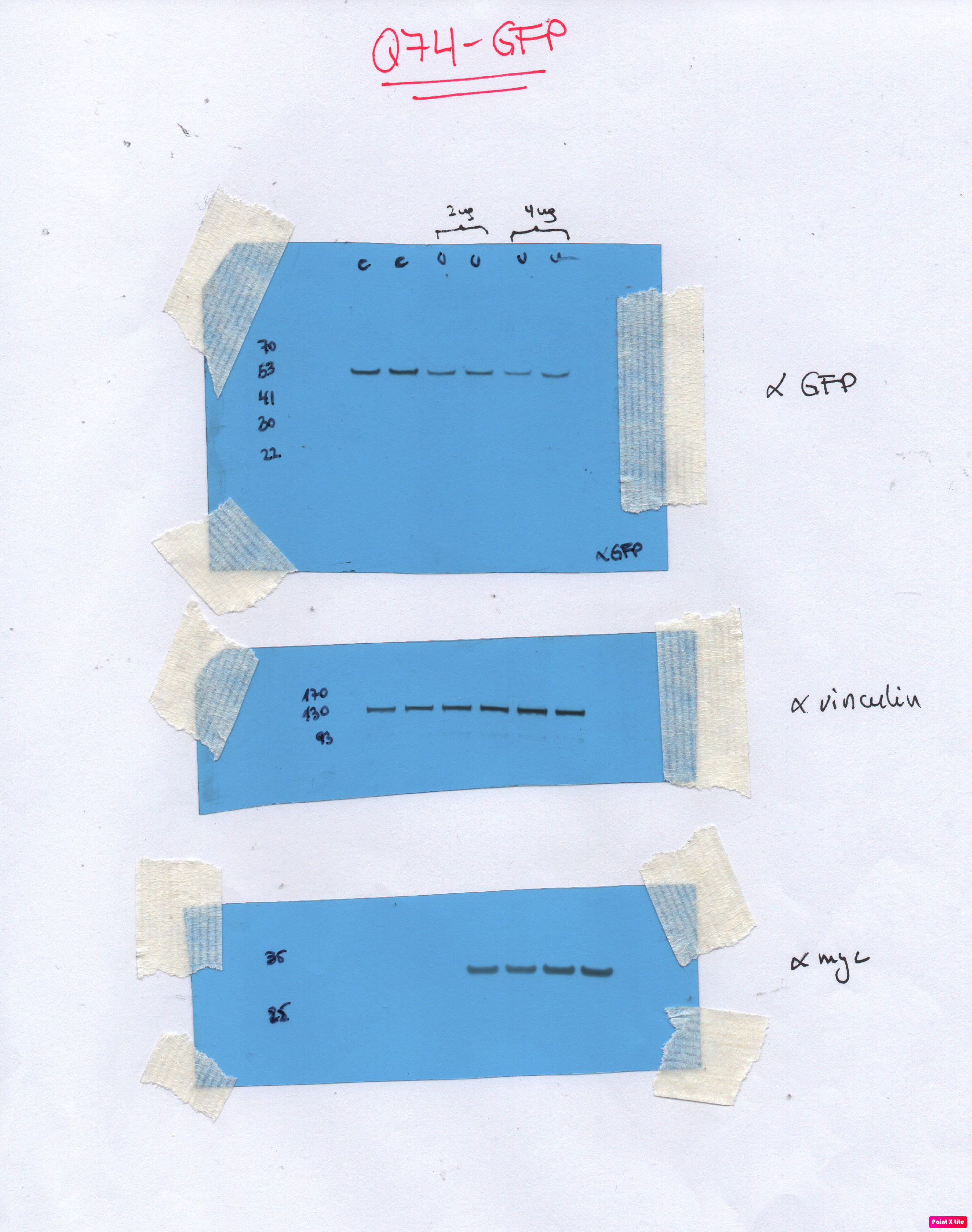

Supplement: Figure 5—source data 1. [file elife-101496-fig5-data1.zip › Figure 5-source data 1/Figure 5D-source data 1.tif]

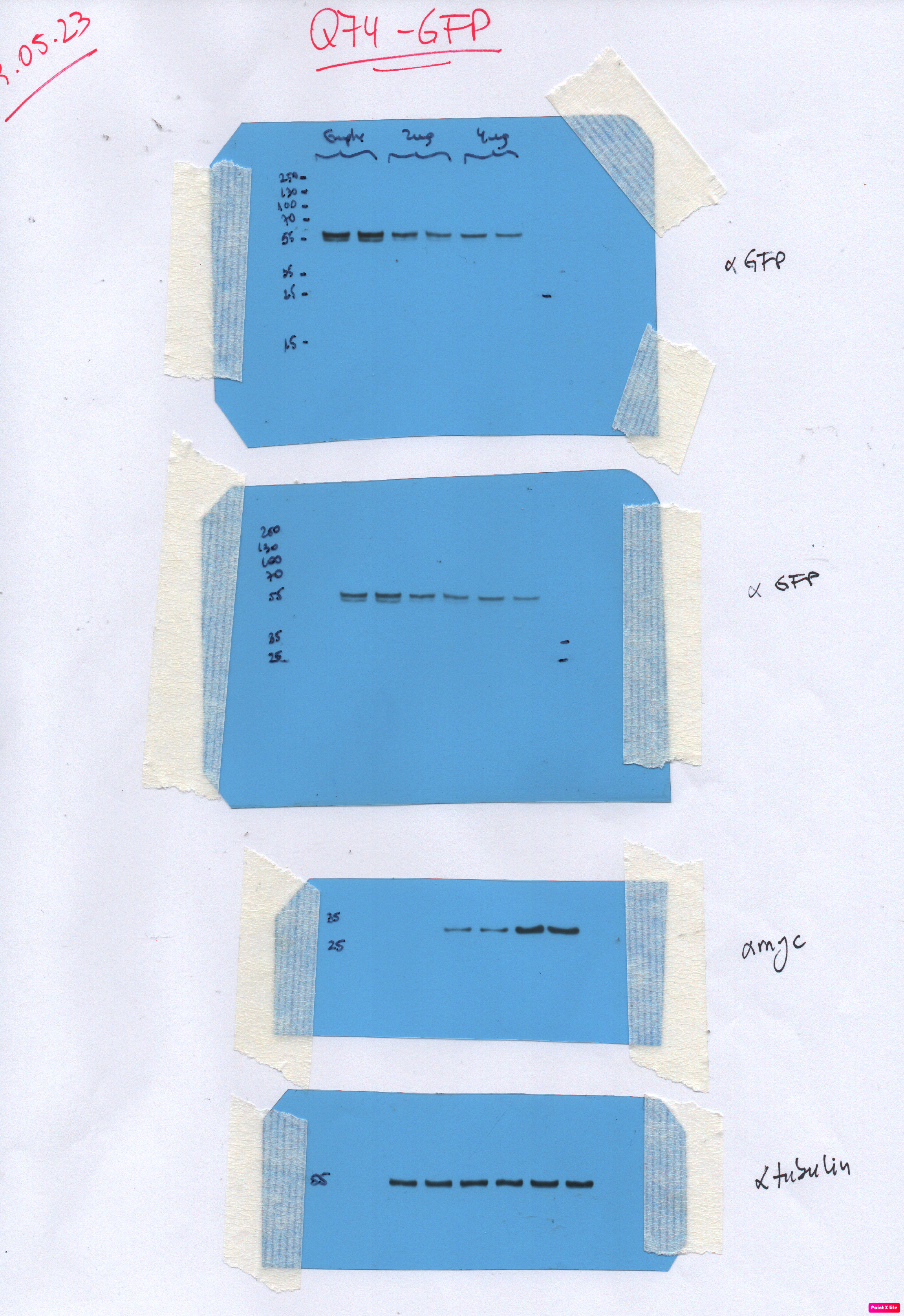

Supplement: Figure 5—source data 1. [file elife-101496-fig5-data1.zip › Figure 5-source data 1/Figure 5D_source data 1.tif]

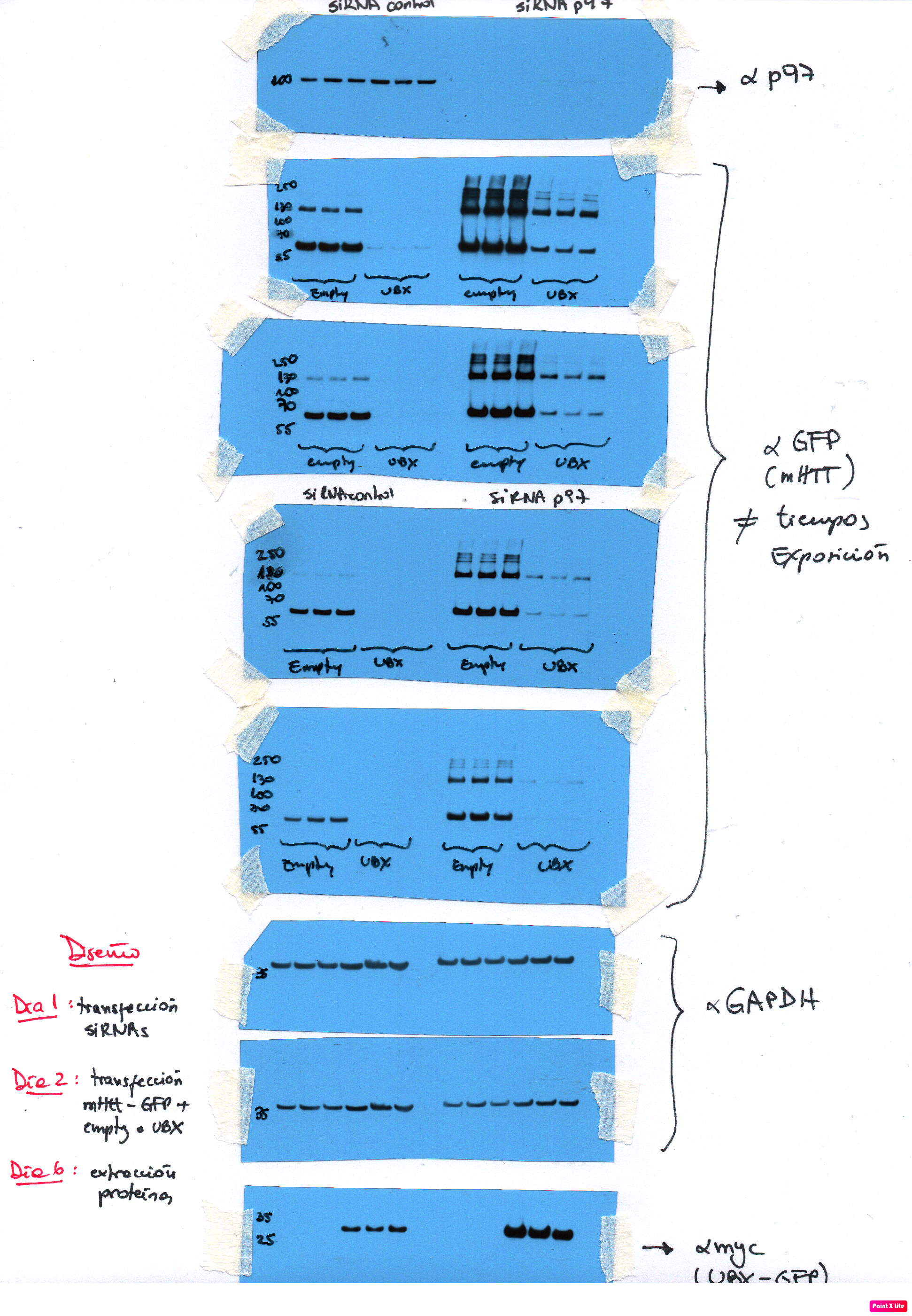

Supplement: Figure 5—figure supplement 1—source data 1. [file elife-101496-fig5-figsupp1-data1.zip › Figure 5-figure supplement 1-data 1/Figure 5-figure supplement 3-source data 1.tif]

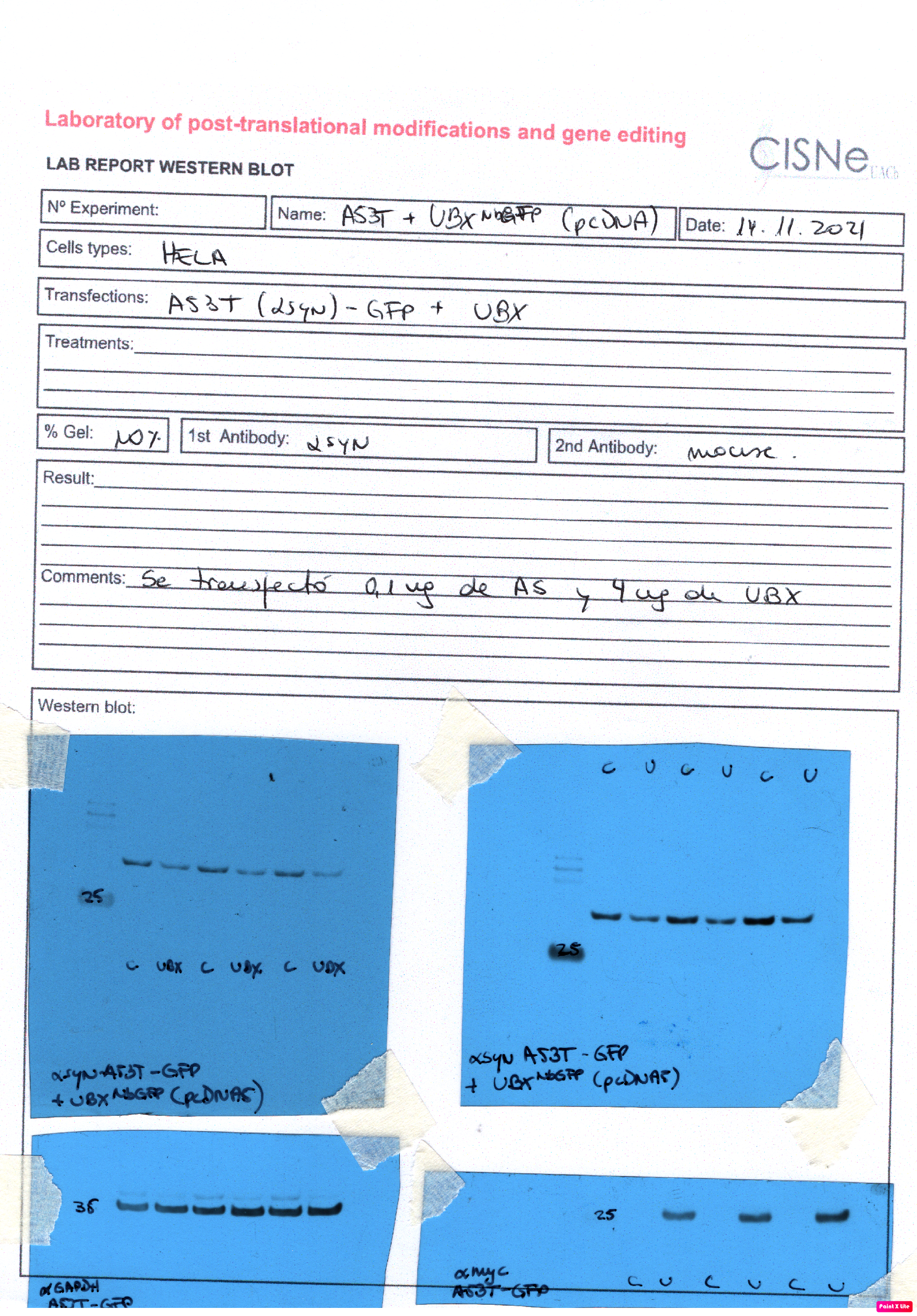

Supplement: Figure 6—source data 1. [file elife-101496-fig6-data1.zip › Figure 6-source data 1/Figure 6A (extra)-source data 1.tif]

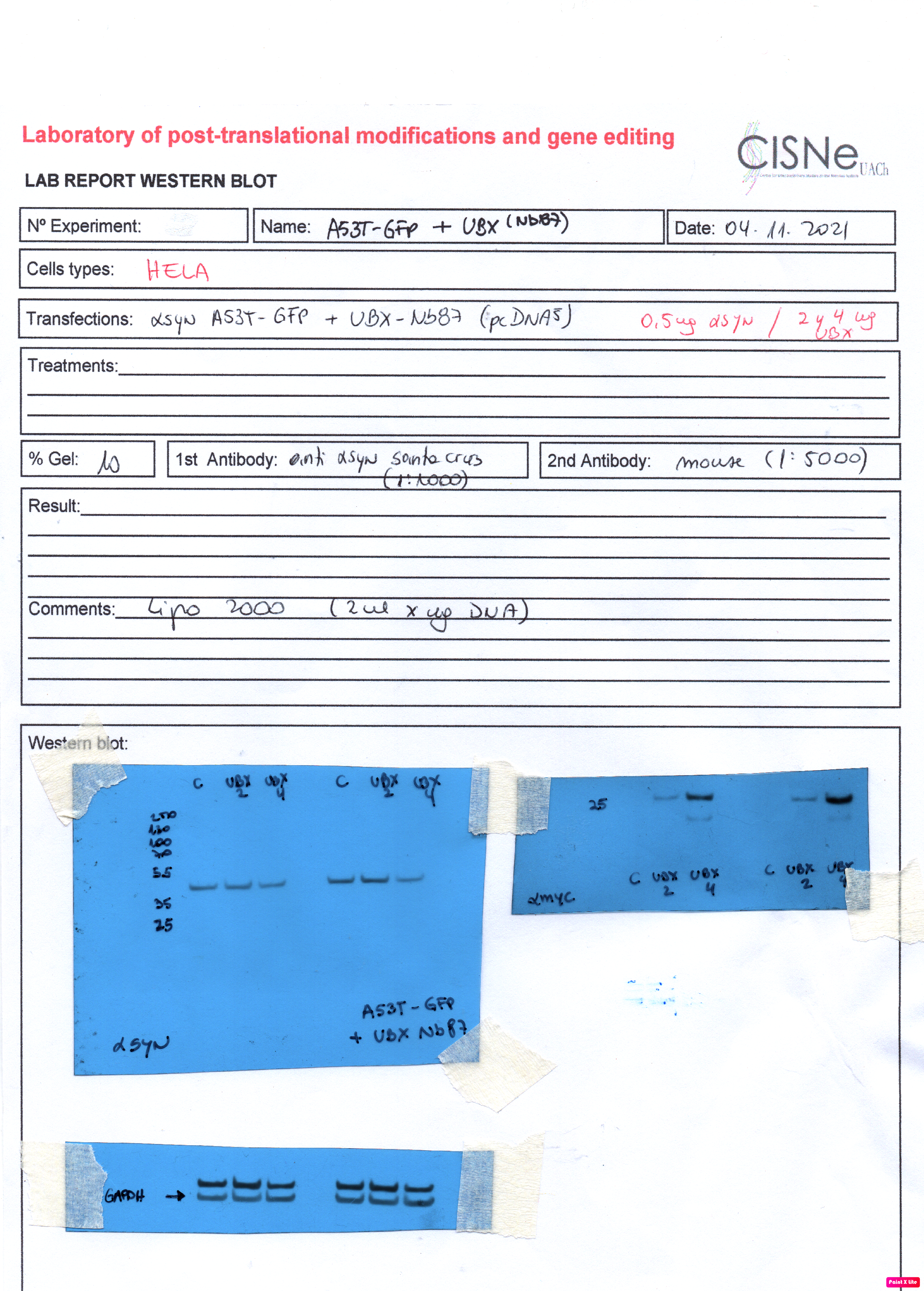

Supplement: Figure 6—source data 1. [file elife-101496-fig6-data1.zip › Figure 6-source data 1/Figure 6A-source data 1.tif]

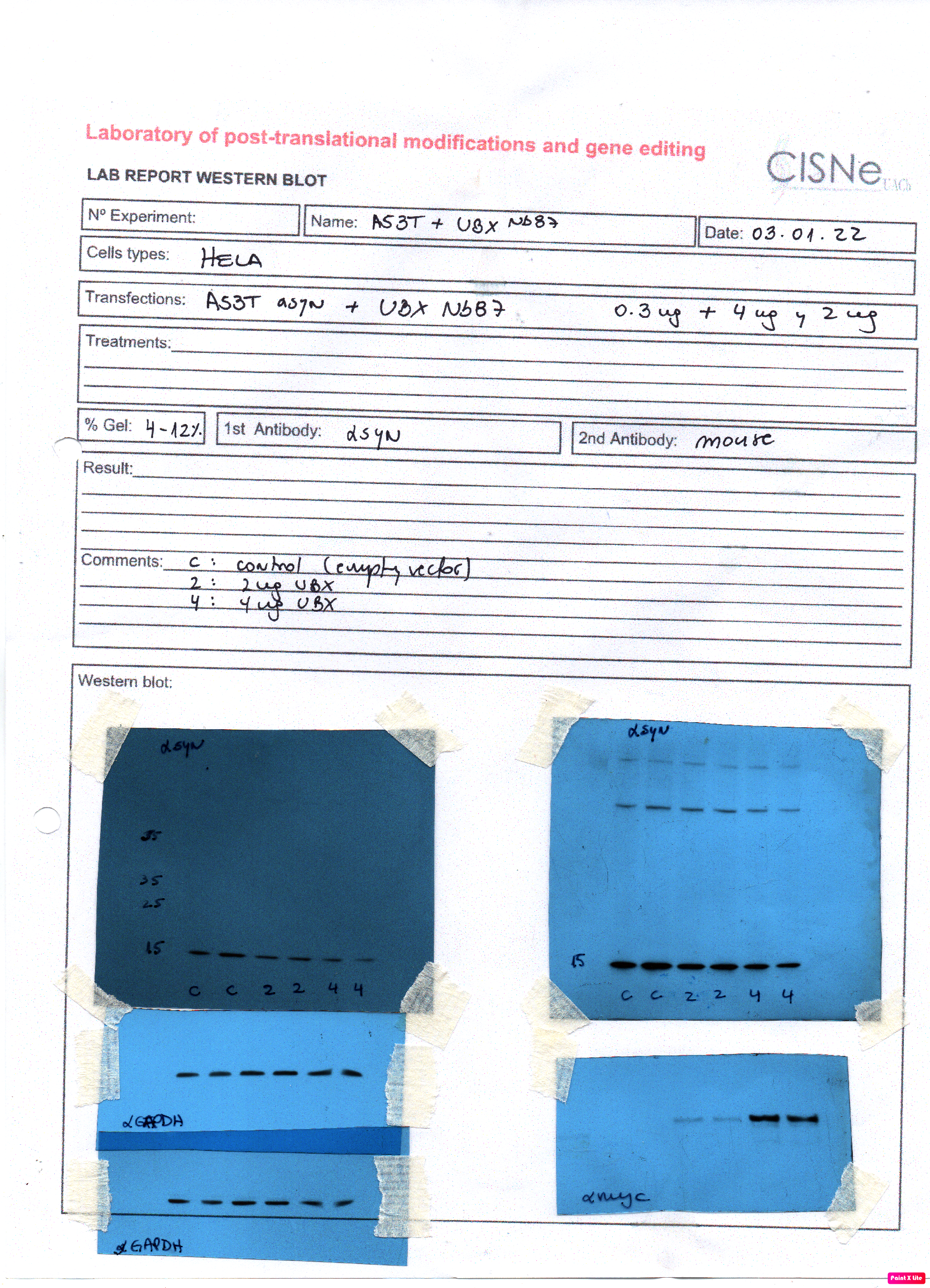

Supplement: Figure 6—source data 1. [file elife-101496-fig6-data1.zip › Figure 6-source data 1/Figure 6C-source data 1.tif]

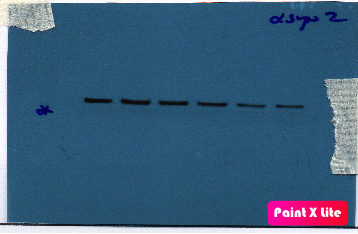

Supplement: Figure 6—source data 1. [file elife-101496-fig6-data1.zip › Figure 6-source data 1/Figure 6C_assay2_AS-source data 1.tif]

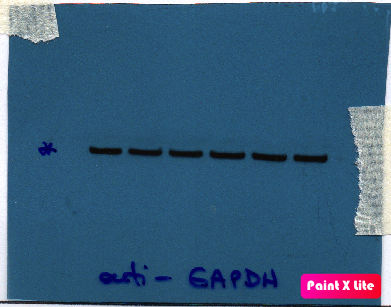

Supplement: Figure 6—source data 1. [file elife-101496-fig6-data1.zip › Figure 6-source data 1/Figure 6C_assay2_GAPDH-source data 1.tif]
