# Supplementary material for: Specific proteolysis mediated by a p97-directed proteolysis-targeting chimera (p97-PROTAC)
Source: eLife. 2025 Nov 26;14:e101496. doi: 10.7554/eLife.101496 (PMC12755880; doi:10.7554/eLife.101496)
Supplement: Figure 1—source data 2. [file elife-101496-fig1-data2.zip › Figure 1-source data 2/Figure 1C-source data 2.pdf]

Twenty micrograms of total protein from cells co-transfected with 0.5 µg of Coilin-GFP and different concentrations of the PROTAC-p97 (2 and 4 µg), or 4 µg of an empty vector, were loaded.

The experiment was performed in duplicate using independent samples. The nitrocellulose membrane was cut at the 35 kDa marker to allow separate incubation of the lower part with **anti-GAPDH antibody**. This section was subsequently stripped and re-probed with anti-Myc tag antibody to detect the expression of the degradation system

The nitrocellulose membrane was incubated with an **anti-GFP antibody** to detect the Coilin-GFP fusion protein

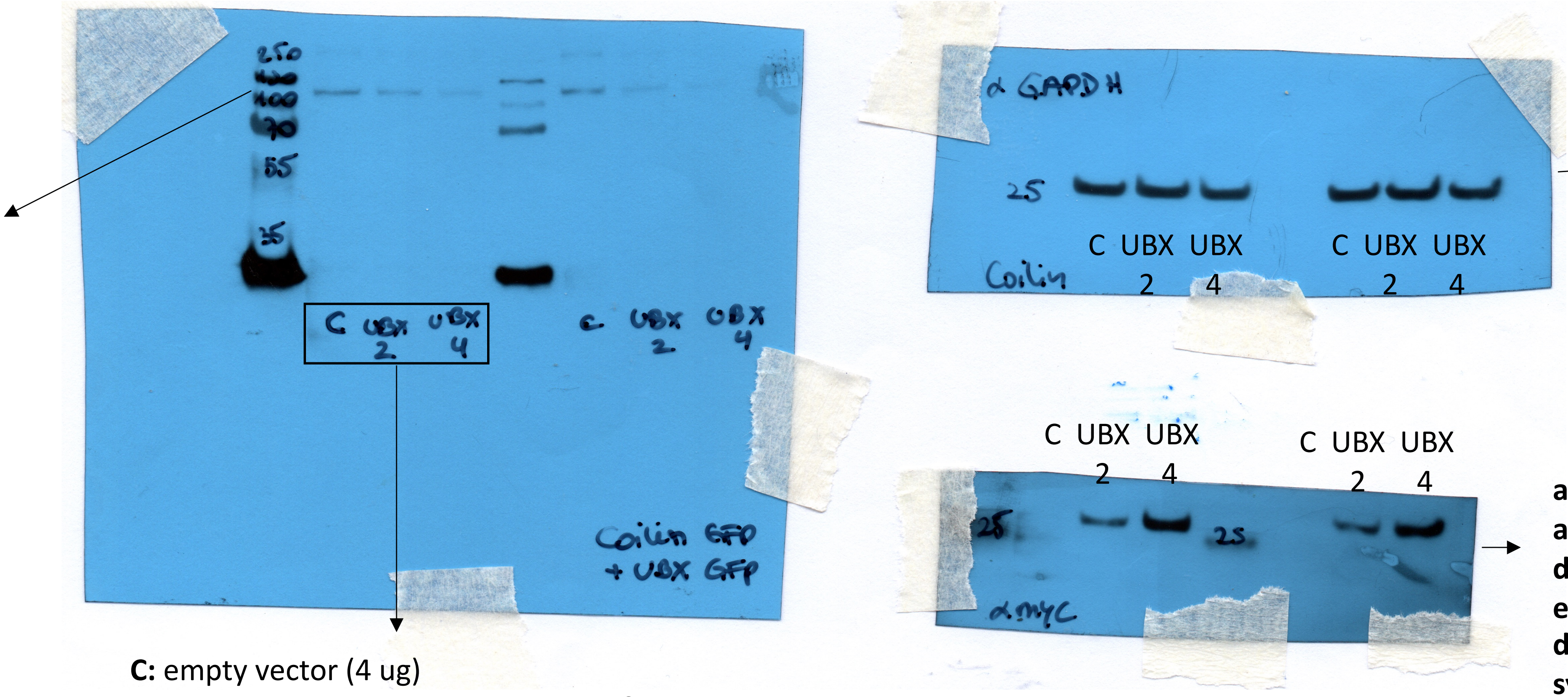

anti-GAPDH antibody

anti-Myc tag antibody to detect the expression of the degradation system p97-PROTAC

C: empty vector (4 ug)

UBX 2 & **UBX 4**: p97-PROTAC (Ubx-Nb^{GFP})
