# Supplementary material for: Specific proteolysis mediated by a p97-directed proteolysis-targeting chimera (p97-PROTAC)
Source: eLife. 2025 Nov 26;14:e101496. doi: 10.7554/eLife.101496 (PMC12755880; doi:10.7554/eLife.101496)

Twenty micrograms of total protein from cells co-transfected with 0.5 µg of ETV-GFP and different concentrations of the PROTAC-p97 (2 and 4 µg), or 4 µg of an empty vector (C), were loaded.

The experiment was performed in duplicate using independent samples. After incubation with anti-GFP antibody to detect the ETV1-GFP protein, the nitrocellulose membrane was cut at the 35 kDa marker. The lower portion was stripped and incubated with anti-GAPDH antibody. To assess the expression of the degradation system, the same membrane was stripped again and re-incubated with an anti-Myc tag antibody.

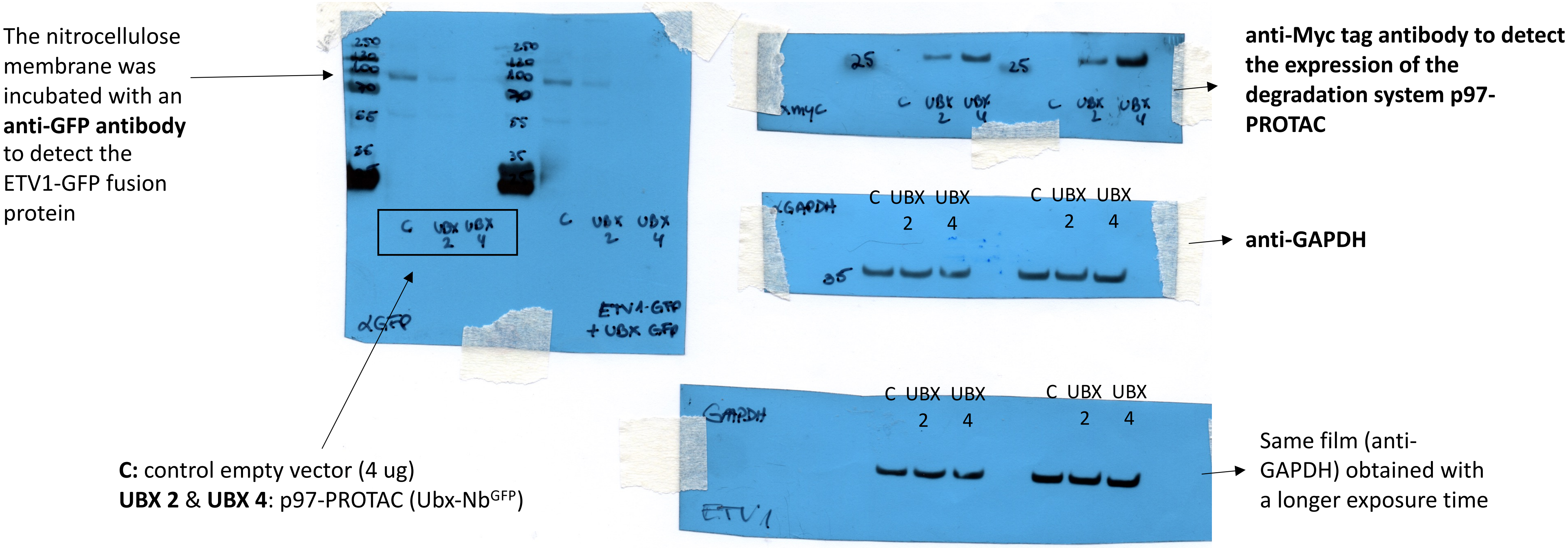

Supplement: Figure 1—source data 2. [file elife-101496-fig1-data2.zip › Figure 1-source data 2/Figure 1G-source data 2.pdf]
