# Supplementary material for: Specific proteolysis mediated by a p97-directed proteolysis-targeting chimera (p97-PROTAC)
Source: eLife. 2025 Nov 26;14:e101496. doi: 10.7554/eLife.101496 (PMC12755880; doi:10.7554/eLife.101496)
Supplement: Figure 1—figure supplement 1—source data 2. [file elife-101496-fig1-figsupp1-data2.zip › Figure 1-figure supplement 1-source data 2/Figure 1-figure supplement 1E-source data 2.pdf]

Twenty micrograms of total protein from cells transfected with 0.5 µg of Coilin-GFP and different concentrations of the PROTAC-p97 using the Nb87 (anti-α-synuclein, used here as a negative control) were loaded. Specifically, cells were transfected **with 2 or 4 µg of p97-PROTAC-Nb87**, or with **4 µg of an empty vector.**

The experiments were performed in duplicate using independent samples. The nitrocellulose membrane was cut at the 35 kDa marker to allow separate incubation of the lower part with **anti-GAPDH antibody**. This section was subsequently stripped and re-probed with **anti-Myc tag antibody** to detect the expression of the degradation system.

The membrane incubated with **anti-GFP antibody** shows Coilin-GFP protein.

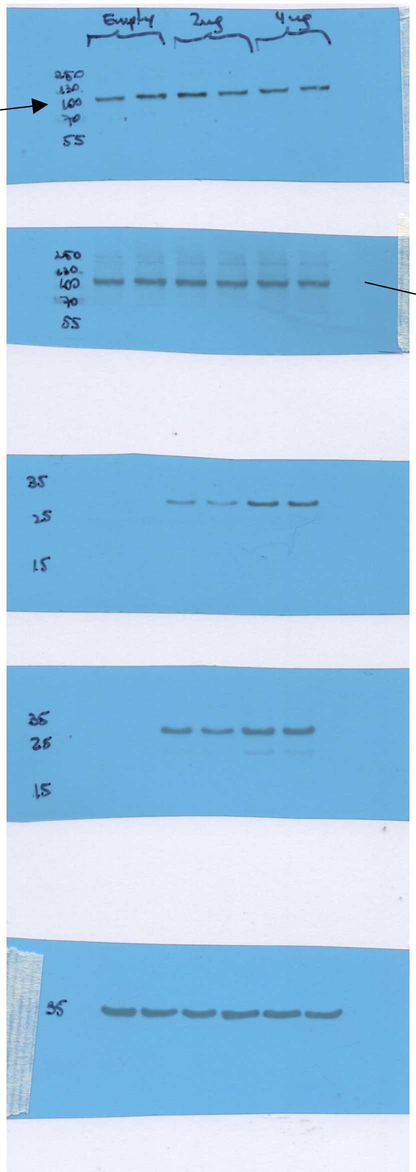

Same film of the membrane incubated with **anti-GFP antibody, but with longer exposure time.**

anti-Myc tag antibody to detect the **expression** of the degradation **system p97-PROTAC.**
The bottom film was exposed for a longer duration.

GAPDH
