# Supplementary material for: Specific proteolysis mediated by a p97-directed proteolysis-targeting chimera (p97-PROTAC)
Source: eLife. 2025 Nov 26;14:e101496. doi: 10.7554/eLife.101496 (PMC12755880; doi:10.7554/eLife.101496)
Supplement: Figure 1—figure supplement 1—source data 2. [file elife-101496-fig1-figsupp1-data2.zip › Figure 1-figure supplement 1-source data 2/Figure 1-figure supplement 1F-source data 2.pdf]

Twenty micrograms of total protein from cells transfected with 0.5 µg of **ETV1-GFP** and different concentrations of the PROTAC-p97 using Nb87 (anti-α-synuclein, used here as a **negative control**) were loaded. The experiments were performed in duplicate using independent samples.

The nitrocellulose membrane was cut to allow separate incubation with **anti-GFP (to detect ETV1-GFP)** and anti-Myc tag antibodies. After developing the signal for ETV1-GFP, the corresponding membrane section was stripped and re-probed with **anti-vinculin** as a loading control.

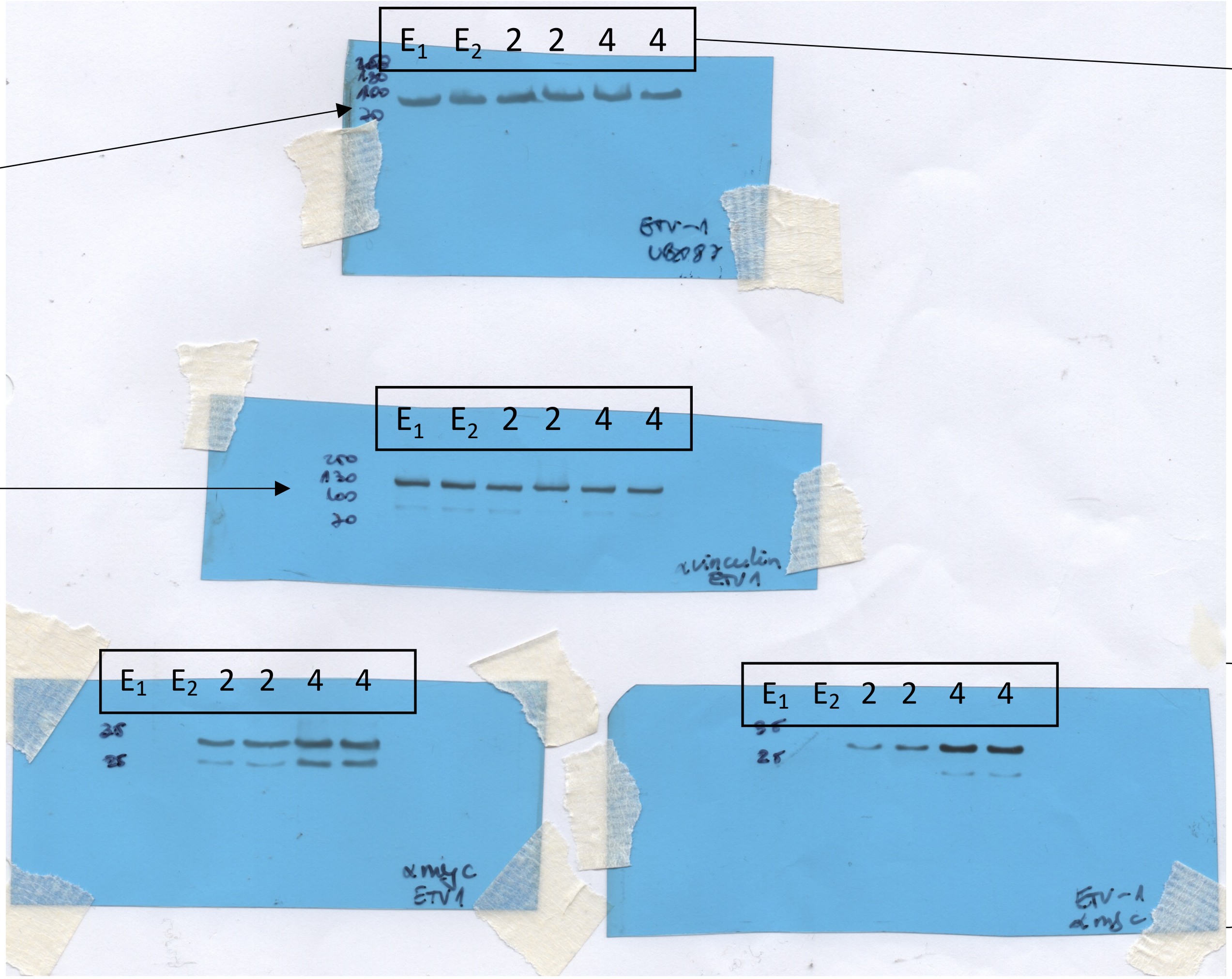

Cells were transfected with **2 or 4 µg of p97-PROTAC-Nb87**, or **with 4 µg of an empty vector (E).**

Both films display expression of the degradation system **(anti-myc-tag).** The film on the left was acquired with a longer exposure time compared to the one on the right.
