# Supplementary material for: Specific proteolysis mediated by a p97-directed proteolysis-targeting chimera (p97-PROTAC)
Source: eLife. 2025 Nov 26;14:e101496. doi: 10.7554/eLife.101496 (PMC12755880; doi:10.7554/eLife.101496)

Twenty micrograms of total protein from cells transfected with 0.5 µg of Emerin-GFP and different concentrations of the PROTAC-p97 using the Nb87 (anti-α-synuclein, used here as a negative control) were loaded. Specifically, cells were transfected with 2 or 4 µg of **p97-PROTAC-Nb87**, or with **4 µg of an Empty vector**. The experiments were performed in duplicate using independent samples

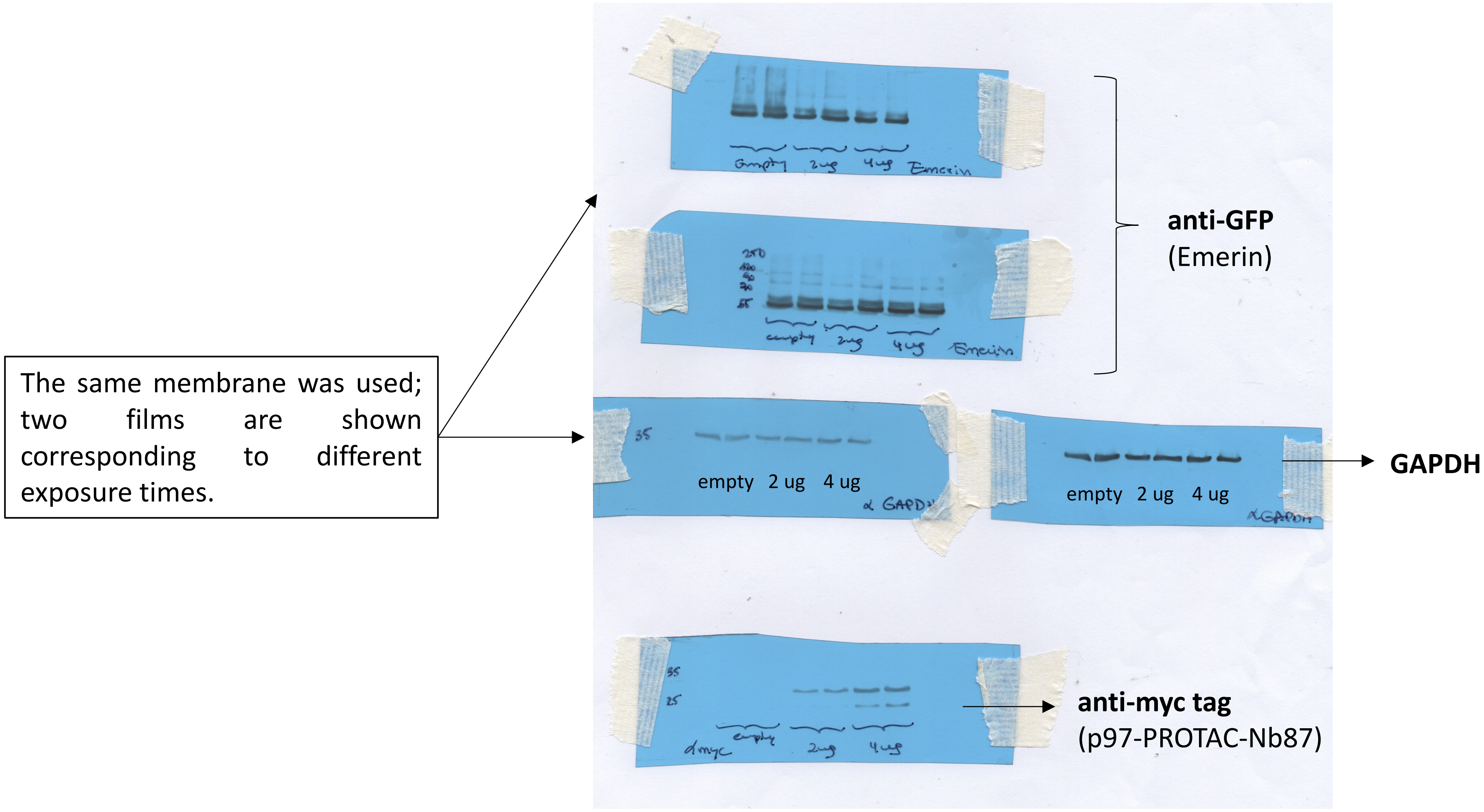

Supplement: Figure 1—figure supplement 1—source data 2. [file elife-101496-fig1-figsupp1-data2.zip › Figure 1-figure supplement 1-source data 2/Figure 1-figure supplement 1G-source data 2.pdf]
