# Supplementary material for: Specific proteolysis mediated by a p97-directed proteolysis-targeting chimera (p97-PROTAC)
Source: eLife. 2025 Nov 26;14:e101496. doi: 10.7554/eLife.101496 (PMC12755880; doi:10.7554/eLife.101496)
Supplement: Figure 2—source data 2. [file elife-101496-fig2-data2.zip › Figure 2-source data 2/Figure 2F-source data 2.pdf]

Forty micrograms of total protein from TP53BP1-YFP knock-in (KI) cells transfected with 4 µg of the PROTAC-p97 Nb-GFP construct or 4 µg of an empty vector were loaded onto a 4–10% gradient polyacrylamide gel. Only the samples loaded with 40 µg of total protein are shown in the manuscript. This experiment was performed in triplicate using independent samples.

C: control empty vector (4 ug DNA)
U: p97-PROTAC (Ubx-Nb^{GFP}) (4 ug DNA)

A film with longer exposure time was required to barely detect the samples loaded with 20 µg of total protein.

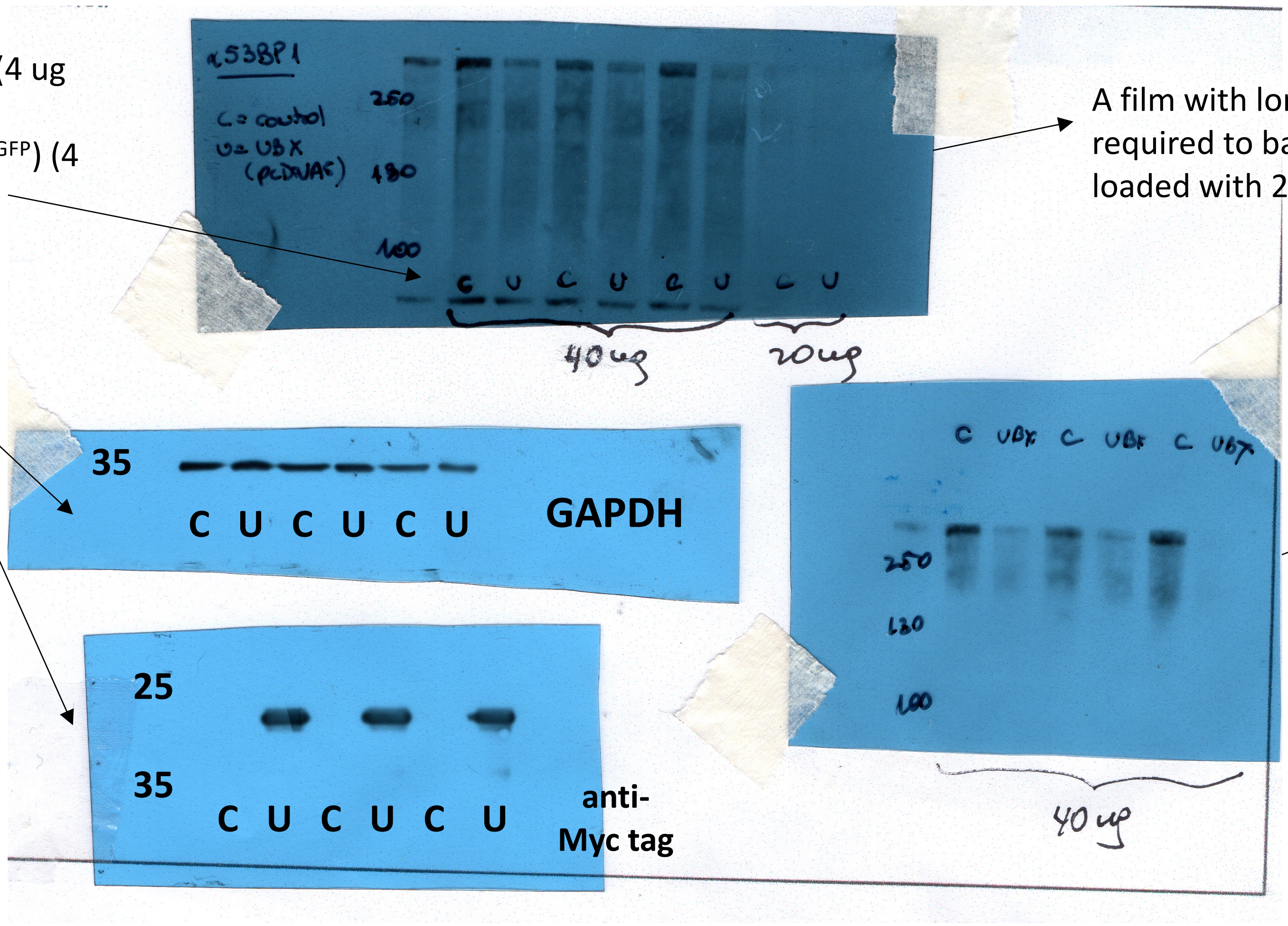

The membrane incubated with **anti-GFP antibody** shows 53BP1-YFP protein only in the samples where 40 µg of total protein were loaded.

anti-Myc tag antibody to detect the expression of the degradation system p97-PROTAC
