# Supplementary material for: Specific proteolysis mediated by a p97-directed proteolysis-targeting chimera (p97-PROTAC)
Source: eLife. 2025 Nov 26;14:e101496. doi: 10.7554/eLife.101496 (PMC12755880; doi:10.7554/eLife.101496)

Twenty micrograms of total protein from cells transfected with 0.5 µg of GFP protein and different concentrations of the PROTAC-p97-Nb^{GFP} (2 and 4 µg), or 4 µg of an empty vector, were loaded. Two polyacrylamide gels were loaded with 20 µg of total protein; both gels were loaded with identical samples. One gel was used for detection with **anti-GFP antibody**, and after stripping, it was re-probed with **anti-Myc tag antibody**. The second gel was used for **GAPDH detection** and, after stripping, was re-probed with anti-GFP to confirm equal protein loading across gels. The experiments were performed in triplicate using independent samples

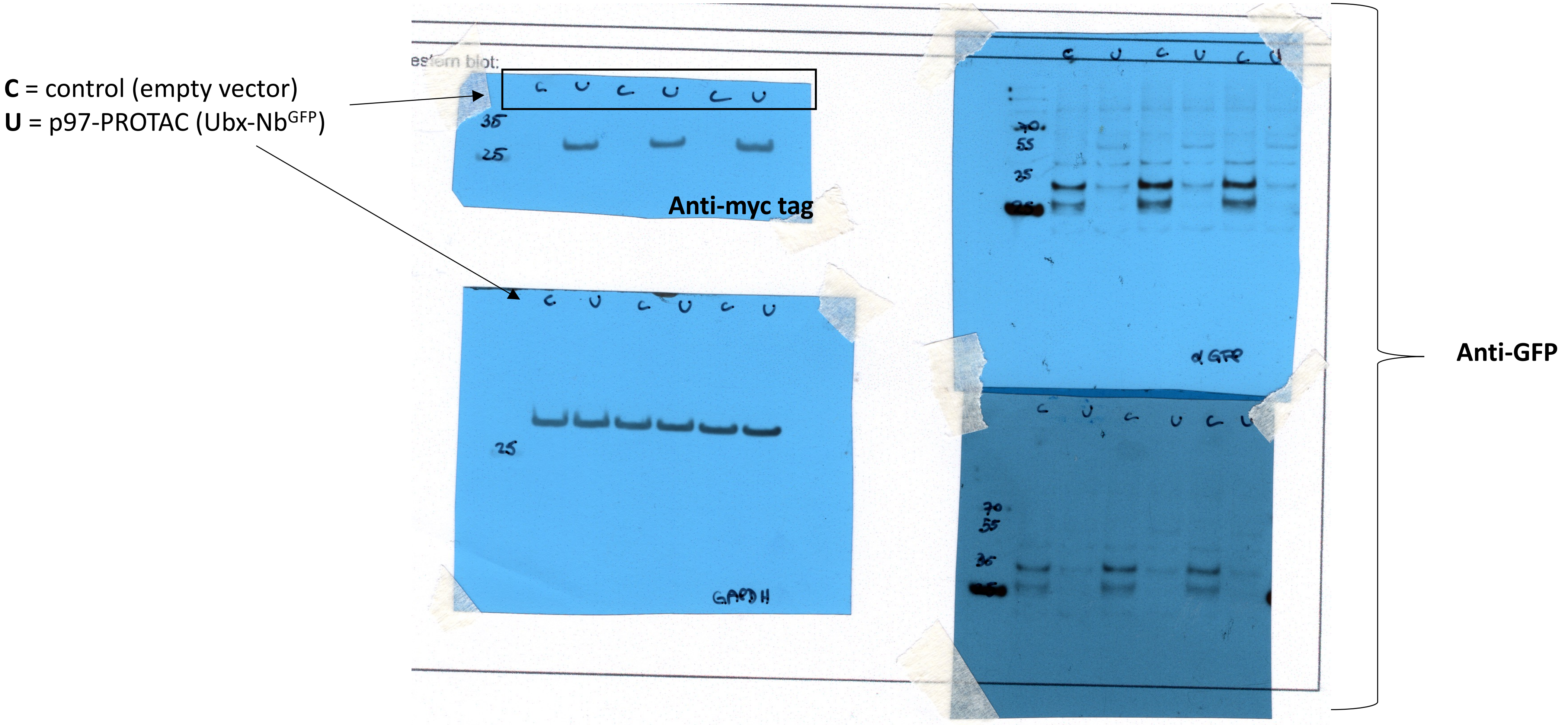

Supplement: Figure 4—source data 2. [file elife-101496-fig4-data2.zip › Figure 4-source data 2/Figure 4C-source data 2.pdf]
