# Supplementary material for: Specific proteolysis mediated by a p97-directed proteolysis-targeting chimera (p97-PROTAC)
Source: eLife. 2025 Nov 26;14:e101496. doi: 10.7554/eLife.101496 (PMC12755880; doi:10.7554/eLife.101496)

Twenty micrograms of total protein from cells co-transfected with 1 µg of a **vector expressing GFP** and 3 µg of the **p97-PROTAC-Ubx-Nb^{GFP} construct (U)**, or 3 µg of an **empty vector (C: control),** were loaded. The following day, cells were treated with the indicated inhibitors for 4 hours, using DMSO as a control. The experiments were performed in duplicate using independent samples

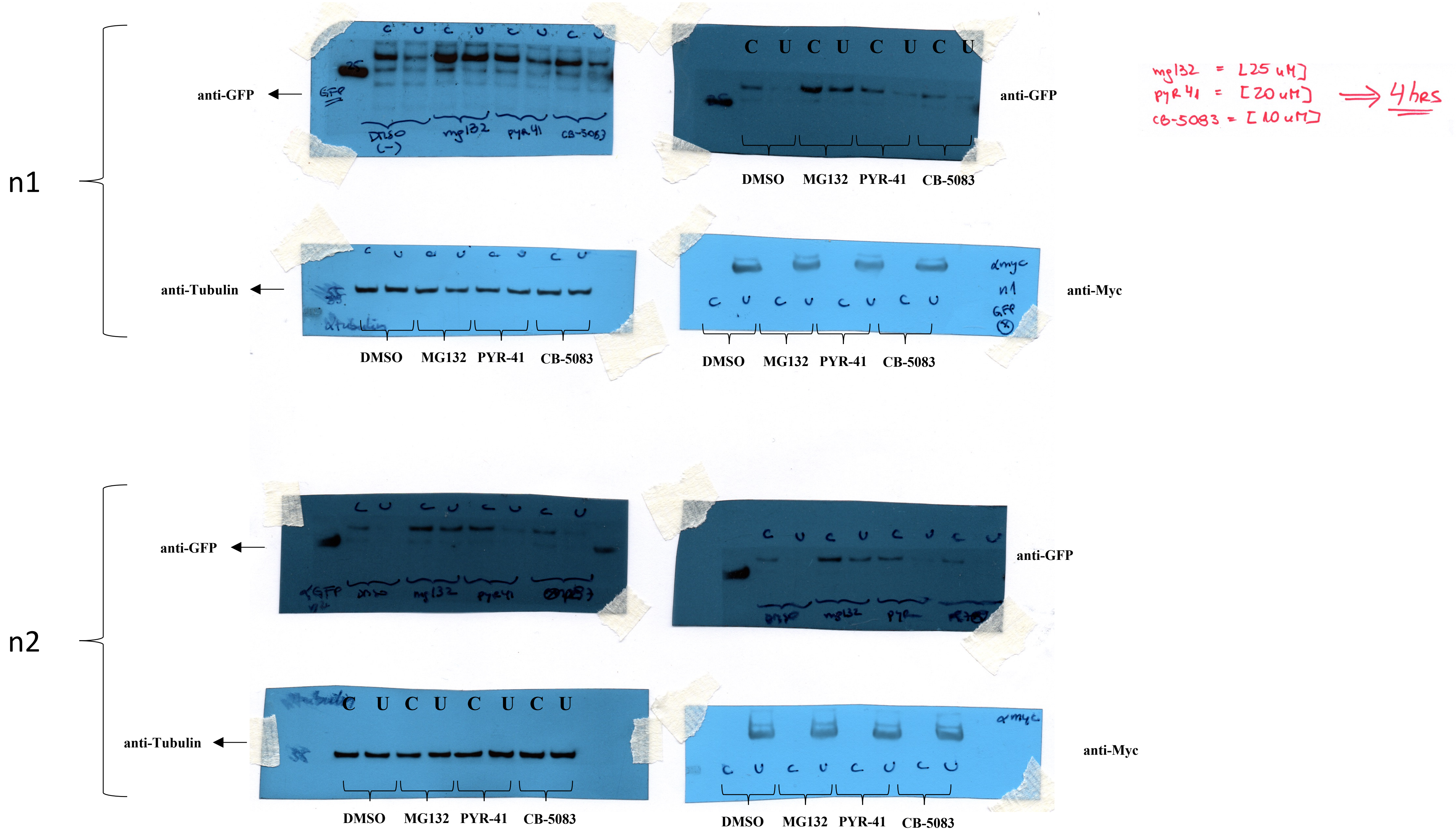

Supplement: Figure 4—source data 2. [file elife-101496-fig4-data2.zip › Figure 4-source data 2/Figure 4E-source data 2.pdf]
