# Supplementary material for: Specific proteolysis mediated by a p97-directed proteolysis-targeting chimera (p97-PROTAC)
Source: eLife. 2025 Nov 26;14:e101496. doi: 10.7554/eLife.101496 (PMC12755880; doi:10.7554/eLife.101496)

On the first day, HeLa cells were transfected with siRNAs targeting either VCP or a non-targeting control. On the following day, the same cells were co-transfected with a vector encoding the Emerin-GFP fusion protein (0.5 µg) and either the PROTAC construct expressing the Ubx-NbGFP domain (4 µg) or an empty vector (4 µg). After 24 hours, total protein was extracted from each well and the samples were analyzed by polyacrylamide gel electrophoresis. Each experiment was performed in triplicate using independent samples.

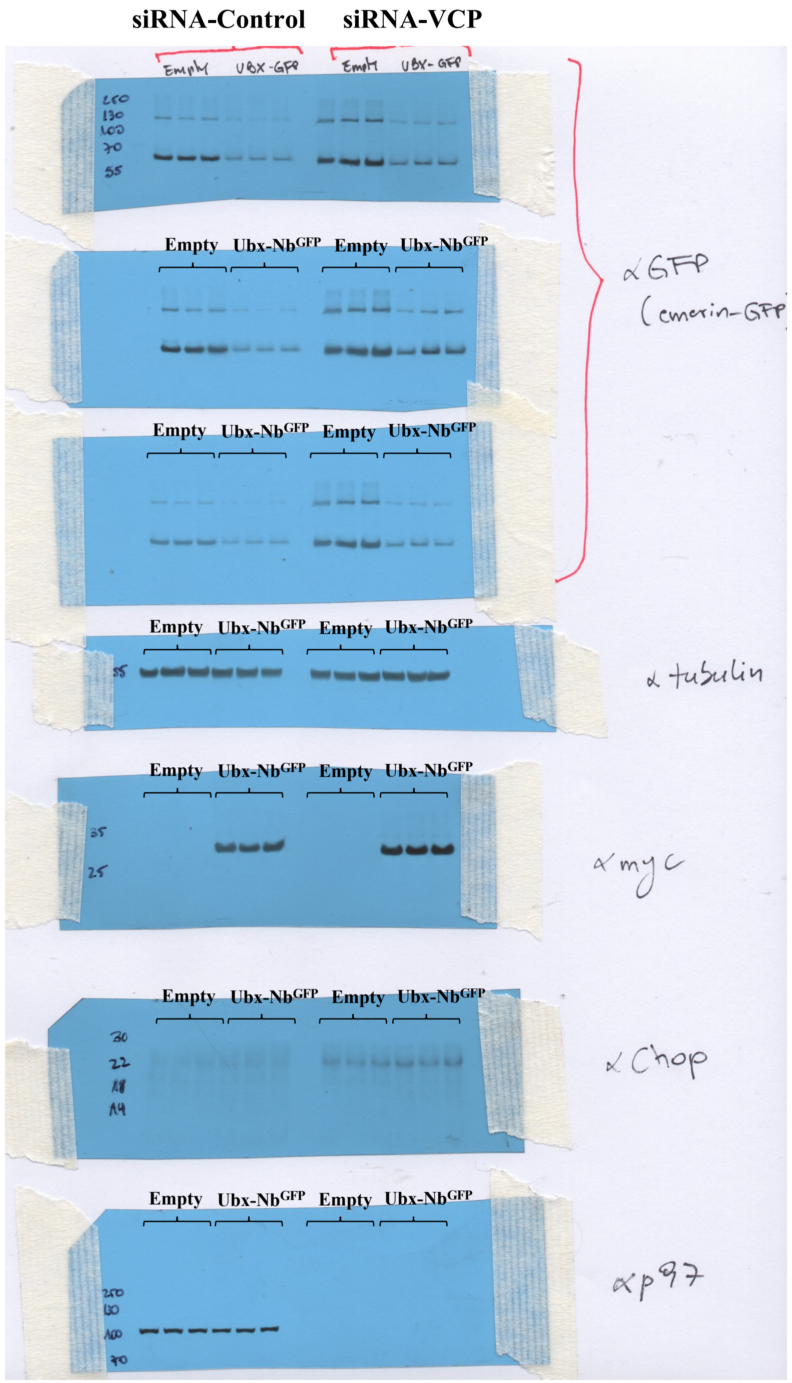

Supplement: Figure 4—source data 2. [file elife-101496-fig4-data2.zip › Figure 4-source data 2/Figure 4G-source data 2.pdf]
