# Supplementary material for: Specific proteolysis mediated by a p97-directed proteolysis-targeting chimera (p97-PROTAC)
Source: eLife. 2025 Nov 26;14:e101496. doi: 10.7554/eLife.101496 (PMC12755880; doi:10.7554/eLife.101496)

HeLa cells were co-transfected with a vector encoding the Emerin-GFP fusion protein (0.5 µg) and either the PROTAC construct expressing the Ubx-Nb^{GFP} (UBX^{GFP}) (4 µg) or an empty vector (4 µg). The day after transfection, cells were treated with the inhibitor PYR-41, using DMSO as a control. In experiments 2 and 3, cells were treated with 50 µM PYR-41 for 4 hours; in experiment 1, the same concentration was used but the treatment lasted 3 hours. Each experiment was performed in triplicate using independent samples.

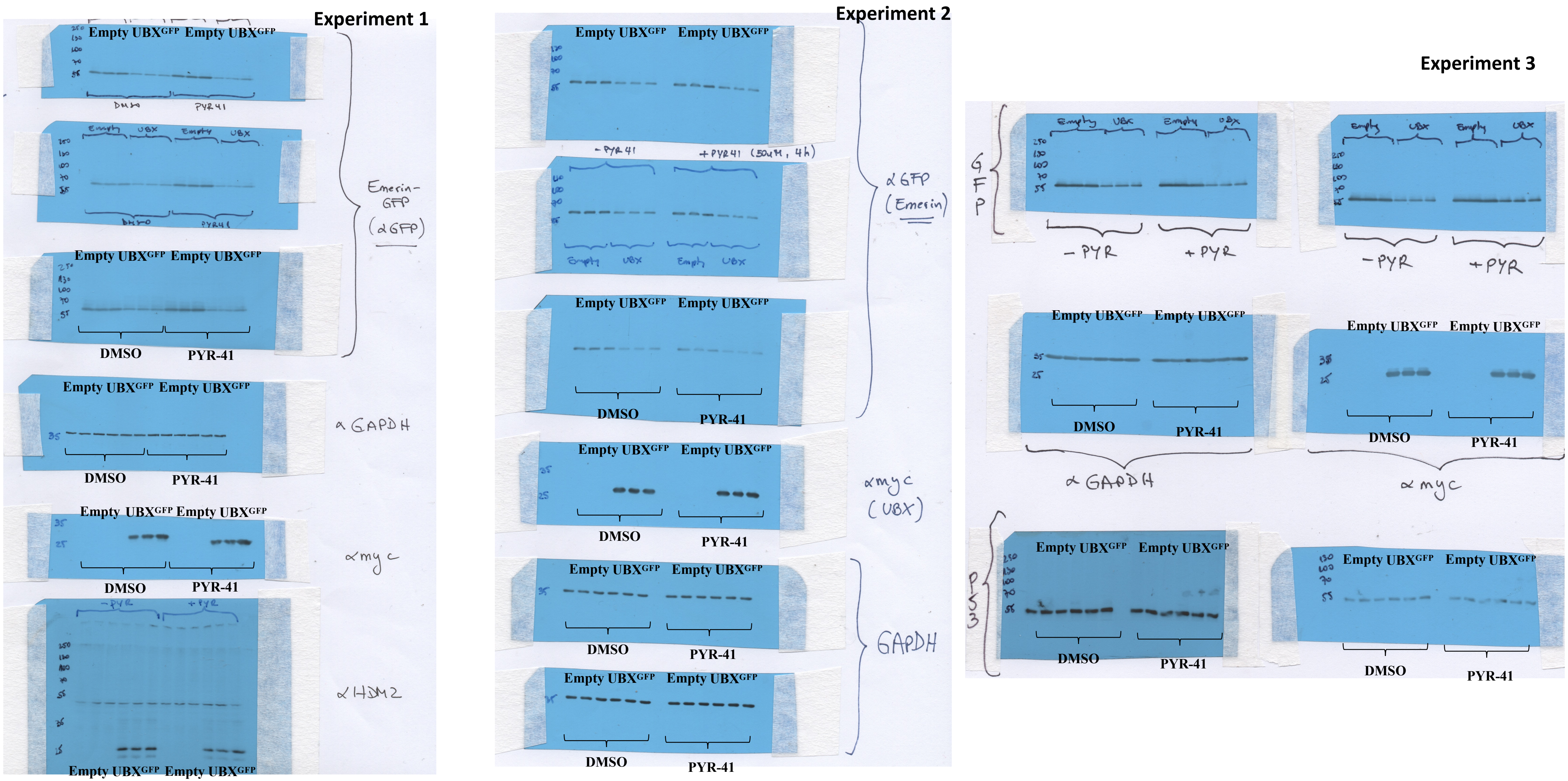

Supplement: Figure 4—source data 2. [file elife-101496-fig4-data2.zip › Figure 4-source data 2/Figure 4J-source data 2.pdf]
