# Supplementary material for: Specific proteolysis mediated by a p97-directed proteolysis-targeting chimera (p97-PROTAC)
Source: eLife. 2025 Nov 26;14:e101496. doi: 10.7554/eLife.101496 (PMC12755880; doi:10.7554/eLife.101496)

HeLa cells were co-transfected with **Emerin-GFP** (0.5 µg) and either an **empty vector** **<u>(E)</u>** or the **p97-PROTAC** construct (**Ubx-Nb^{GFP}, referred to as <u>U</u>)**. The day after transfection, cells were treated for 6 hours with the p97 inhibitor CB-5083 at 4 or 20 µM, or with DMSO as a negative control.

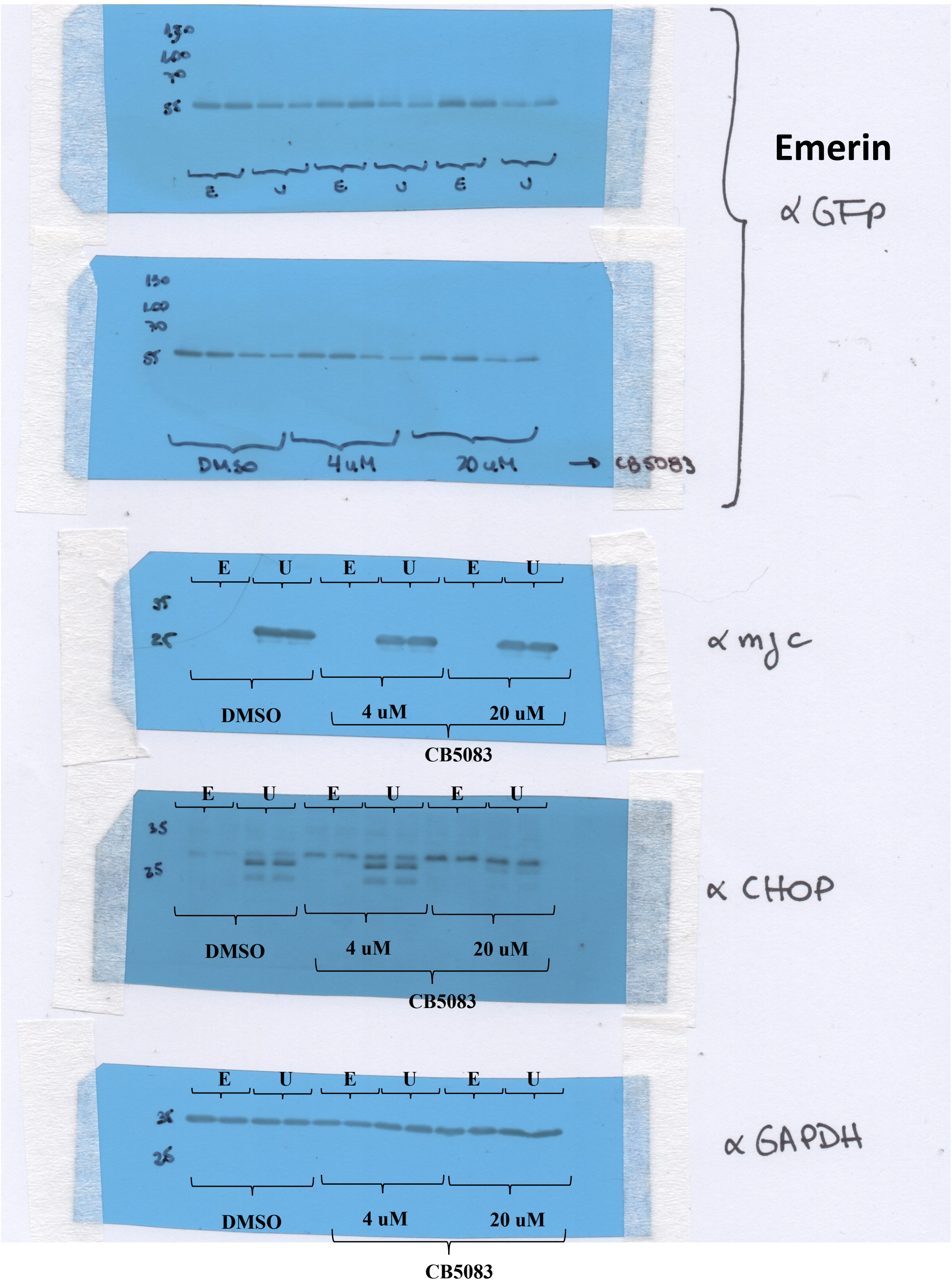

Supplement: Figure 4—source data 2. [file elife-101496-fig4-data2.zip › Figure 4-source data 2/Figure 4L-source data 2.pdf]
