# Supplementary material for: Specific proteolysis mediated by a p97-directed proteolysis-targeting chimera (p97-PROTAC)
Source: eLife. 2025 Nov 26;14:e101496. doi: 10.7554/eLife.101496 (PMC12755880; doi:10.7554/eLife.101496)

HeLa cells were co-transfected **with 0.5 µg of the Nb^{GFP}-Myc-tag** vector and either **2 µg or 4 µg of the VCP/p97-GFP vector**, or an **empty vector (control).** Fifteen micrograms of total protein were loaded. The experiment was performed in duplicate using independent biological samples.

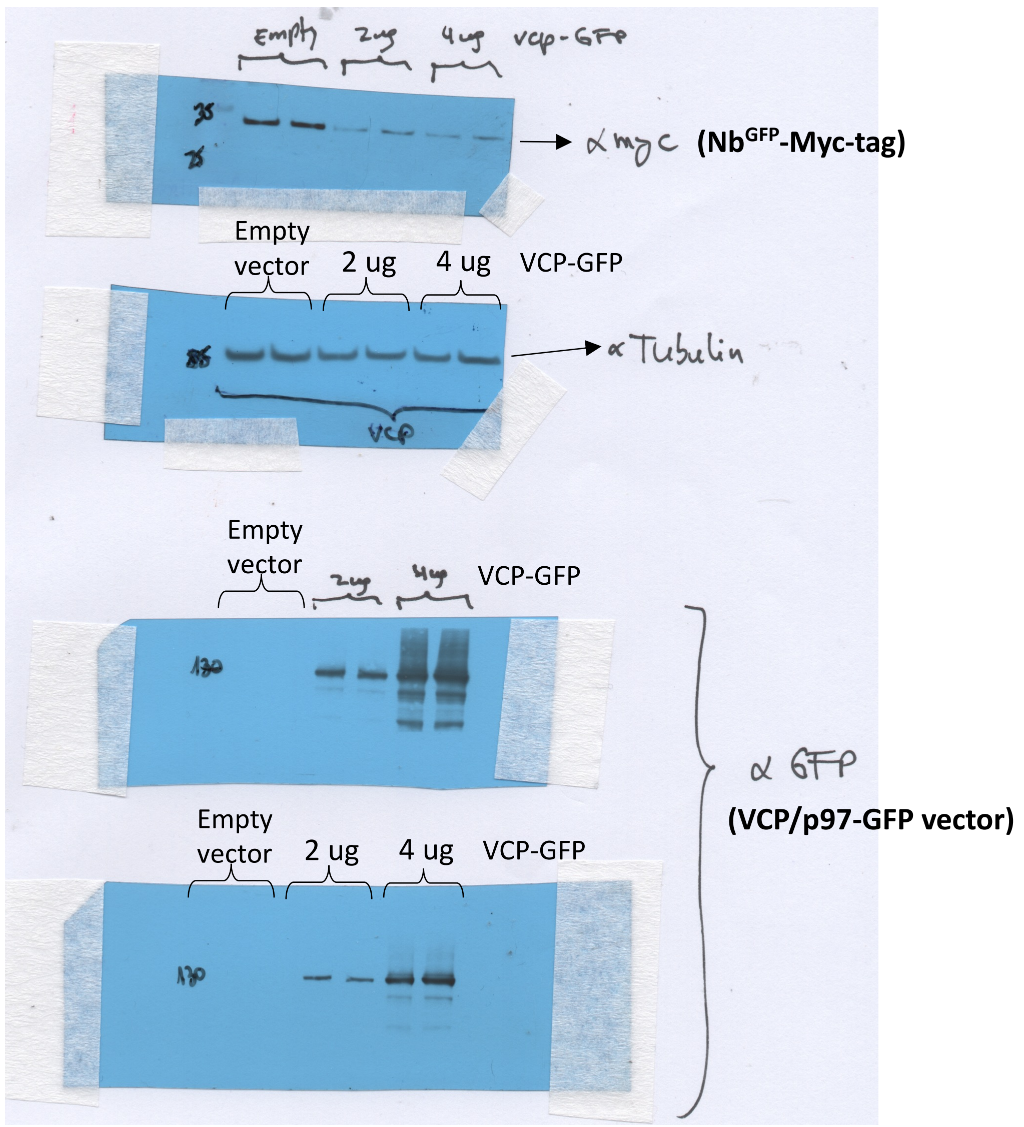

Supplement: Figure 4—figure supplement 1—source data 2. [file elife-101496-fig4-figsupp1-data2.zip › Figure 4-figure supplement 1-source data 2/Figure 4-figure supplement 1A-source data 2.pdf]
