# Supplementary material for: Specific proteolysis mediated by a p97-directed proteolysis-targeting chimera (p97-PROTAC)
Source: eLife. 2025 Nov 26;14:e101496. doi: 10.7554/eLife.101496 (PMC12755880; doi:10.7554/eLife.101496)

Two gels were run, each loaded with 20 µg of total protein from wild-type HeLa cells treated for 4 hours with PYR-41 or DMSO (negative control). One gel was subsequently used for ubiquitin detection, and the other for p53 detection. Subsequently, the membranes were stripped and used to detect loading controls: GAPDH on the membrane previously used for p53 detection, and tubulin on the membrane used for ubiquitin detection.

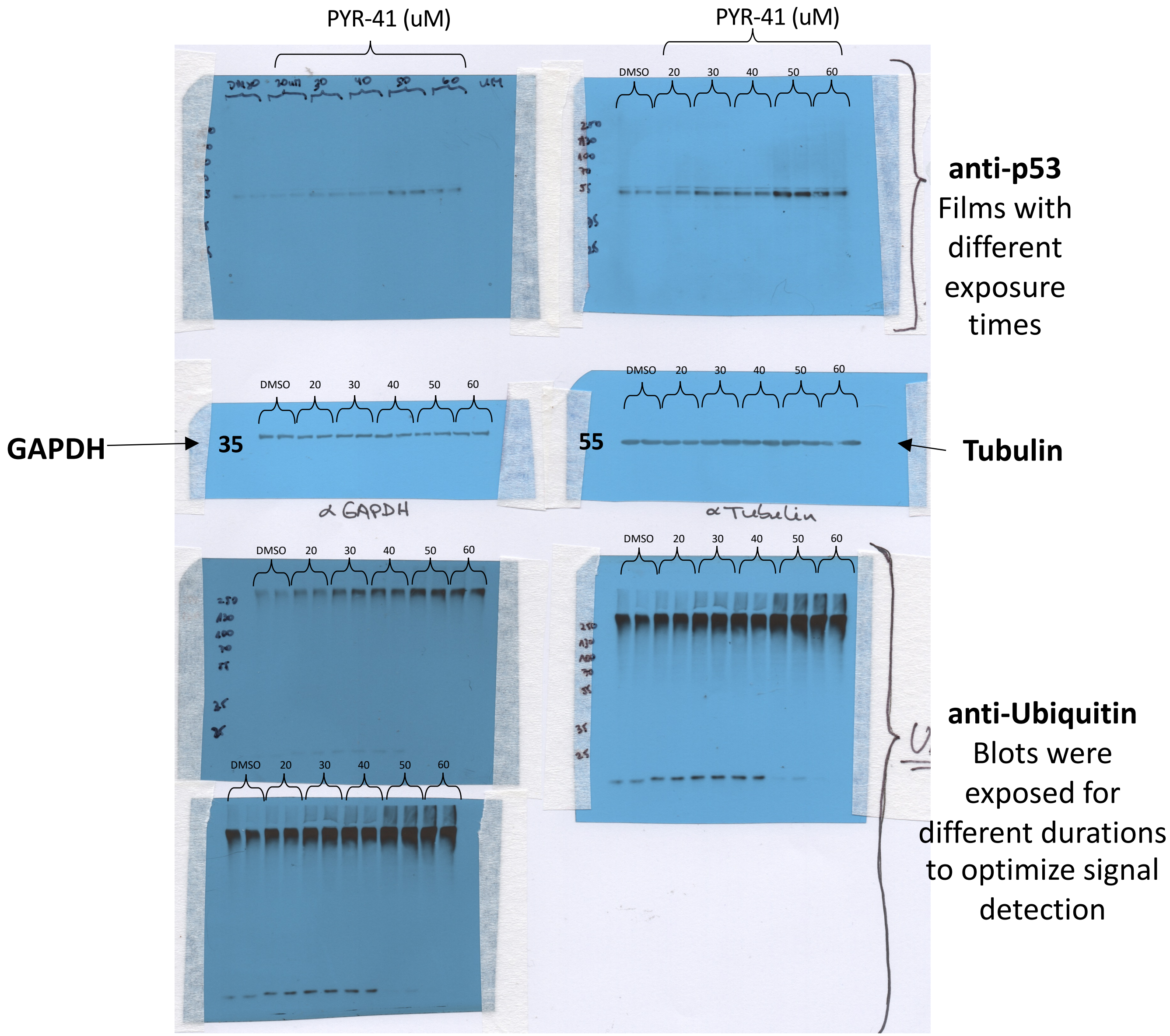

Supplement: Figure 4—figure supplement 1—source data 2. [file elife-101496-fig4-figsupp1-data2.zip › Figure 4-figure supplement 1-source data 2/Figure 4-figure supplement 1C-source data 2.pdf]
