# Supplementary material for: Specific proteolysis mediated by a p97-directed proteolysis-targeting chimera (p97-PROTAC)
Source: eLife. 2025 Nov 26;14:e101496. doi: 10.7554/eLife.101496 (PMC12755880; doi:10.7554/eLife.101496)

HeLa cells were co-transfected with **Q23-GFP (0.5 µg DNA)** and either an **empty vector (E)** or the **p97-PROTAC-Ubx-Nb^{GFP} construct (2 or 4 µg** DNA). The nitrocellulose membrane was cut at the 70 kDa marker to allow separate incubation: one part with **anti-GFP antibody to detect Q23-GFP** expression, and the other with anti-vinculin as a loading control. Subsequently, the membrane previously incubated with anti-GFP was stripped and re-probed with anti-Myc tag antibody to detect expression of the degradation system. The experiment was performed in duplicate using independent biological samples.

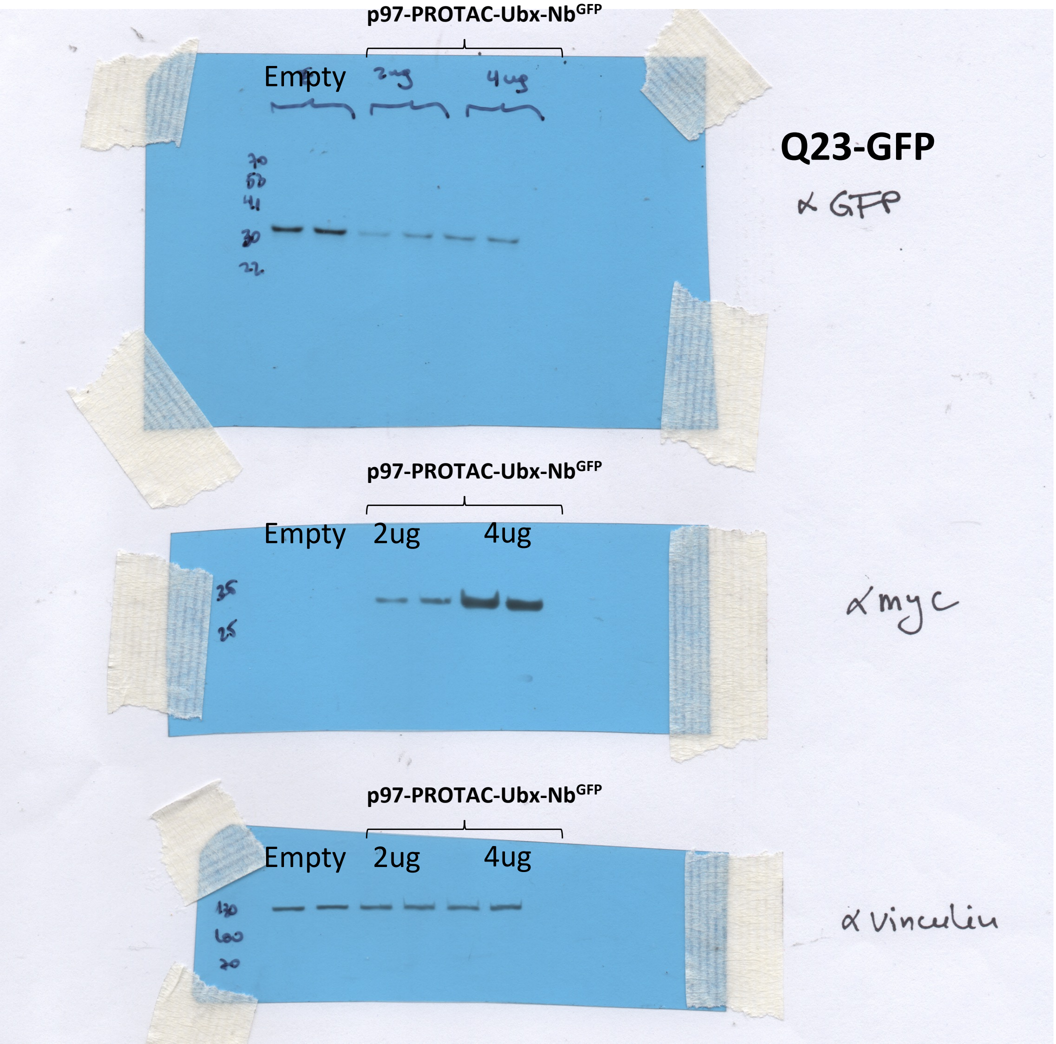

Supplement: Figure 5—source data 2. [file elife-101496-fig5-data2.zip › Figure 5-source data 2/Figure 5A-source data 2.pdf]
