# Supplementary material for: Specific proteolysis mediated by a p97-directed proteolysis-targeting chimera (p97-PROTAC)
Source: eLife. 2025 Nov 26;14:e101496. doi: 10.7554/eLife.101496 (PMC12755880; doi:10.7554/eLife.101496)

HeLa cells were co-transfected with **Q74-GFP (0.5 µg DNA)** and either an **empty vector (C)** or the **p97-PROTAC-Ubx-Nb^{GFP} (U) construct (2 or 4 µg** DNA). Two independent experiments were performed, each conducted in duplicate using independent samples. In both experiments, 20 µg of total protein from co-transfected cells were used. In the first experiment, the entire nitrocellulose membrane was initially incubated with **anti-GFP antibody to detect Q74-GFP**. The membrane was then stripped and cut to allow separate incubation with anti-tubulin and anti-Myc antibodies. In the second experiment, the membrane was cut prior to incubation: one portion was incubated with anti-vinculin and the other with anti-GFP..

Experiment 1

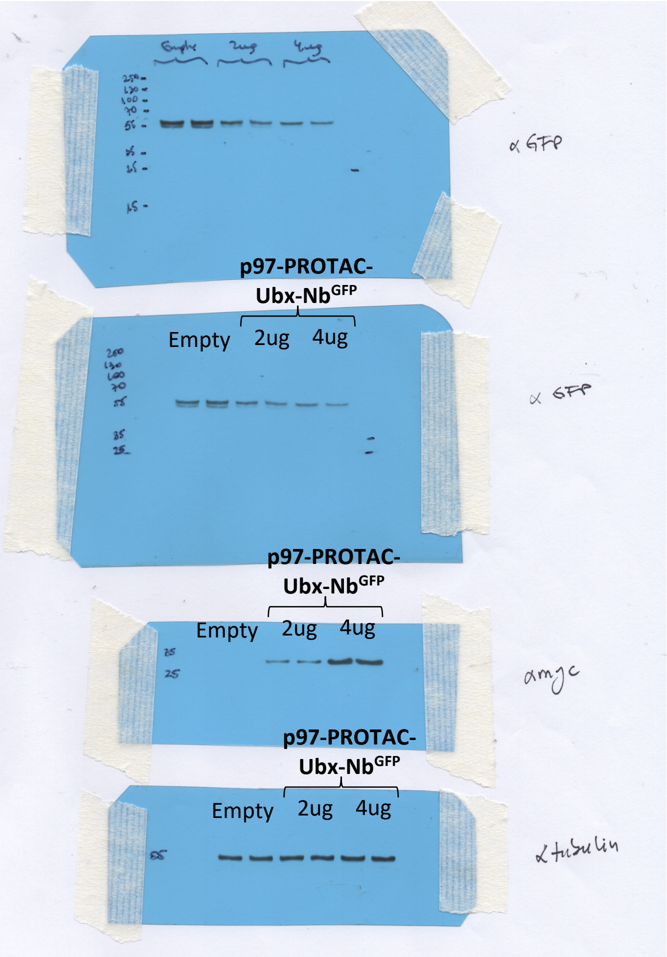

Experiment 2

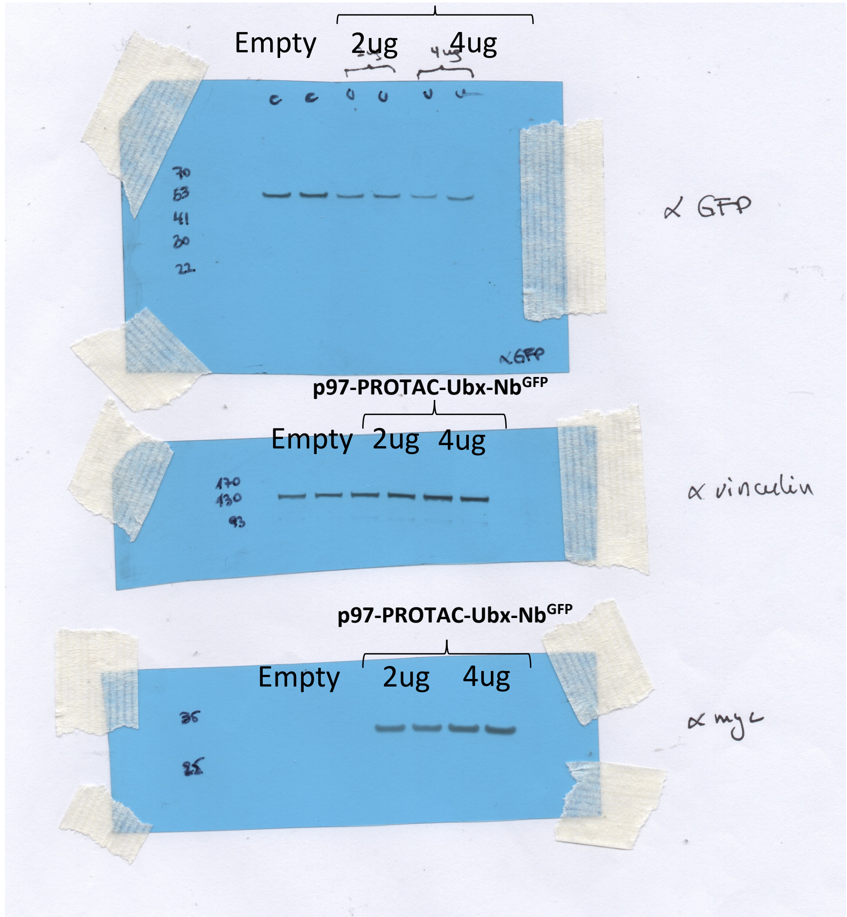

Supplement: Figure 5—source data 2. [file elife-101496-fig5-data2.zip › Figure 5-source data 2/Figure 5D-source data 2.pdf]
