# Supplementary material for: Specific proteolysis mediated by a p97-directed proteolysis-targeting chimera (p97-PROTAC)
Source: eLife. 2025 Nov 26;14:e101496. doi: 10.7554/eLife.101496 (PMC12755880; doi:10.7554/eLife.101496)

HeLa cells were transfected with siRNAs targeting either **VCP (siRNA p97)** or a **non-targeting control (siRNA control).** On the following day, cells were co-transfected with a vector expressing the **Huntingtin Q24-GFP** fusion protein, together with either an **empty vector** or the **p97-PROTAC-Ubx-Nb^{GFP} construct**. The membrane incubated with anti-GFP antibody was developed at **different exposure times**, resulting in multiple films; this approach was used to allow **detection of protein aggregates**. The membrane incubated with **anti-Myc antibody shows expression of the degradation system**, which is increased upon VCP/p97 knockdown with siRNA.

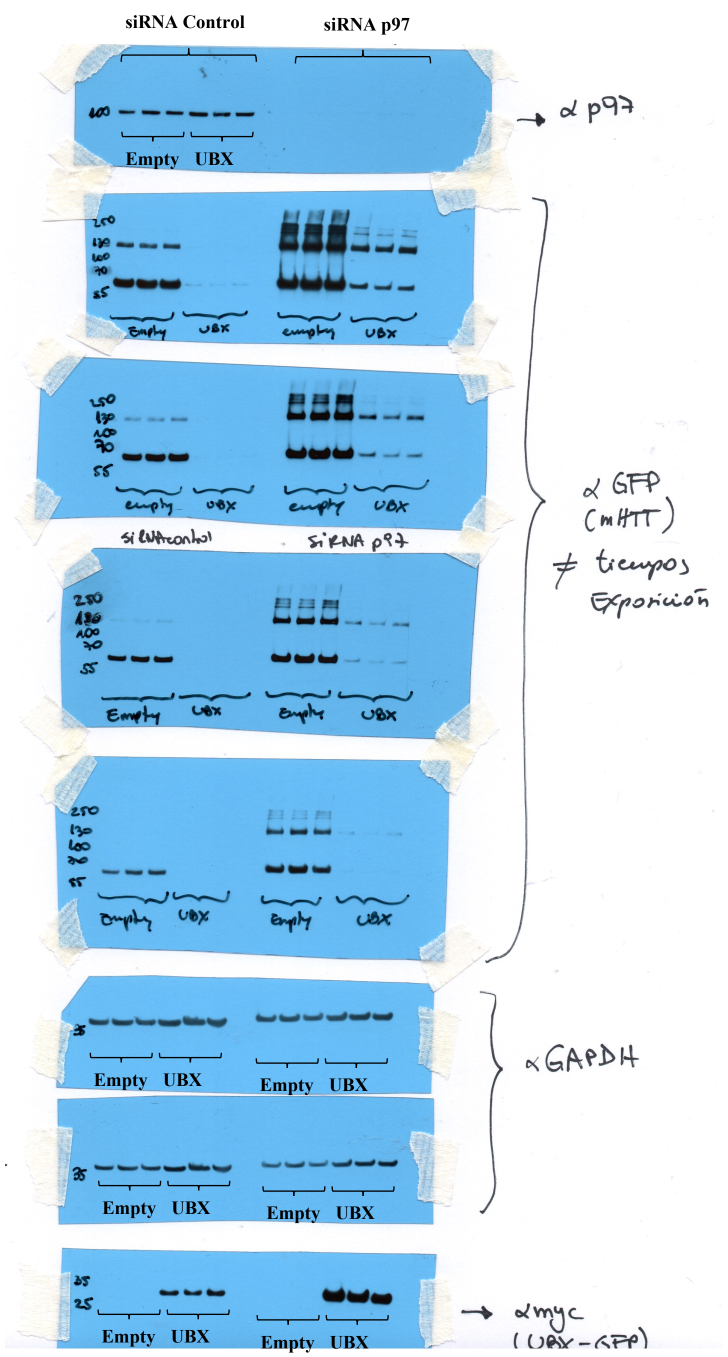

Supplement: Figure 5—figure supplement 1—source data 2. [file elife-101496-fig5-figsupp1-data2.zip › Figure 5-figure supplement 1-data 2/Figure 5-figure supplement 3-source data 2.pdf]
