# Supplementary material for: Specific proteolysis mediated by a p97-directed proteolysis-targeting chimera (p97-PROTAC)
Source: eLife. 2025 Nov 26;14:e101496. doi: 10.7554/eLife.101496 (PMC12755880; doi:10.7554/eLife.101496)

Twenty micrograms of total protein extracted from HeLa cells co-transfected with 0.5 µg of **α-synuclein A53T-GFP** and either 2 or 4 µg of the **p97-PROTAC construct (using the Nb87 nanobody against α-synuclein)**, or with 4 µg of an empty vector as control **(C)**, were loaded. The experiment was performed in duplicate using independent biological samples.

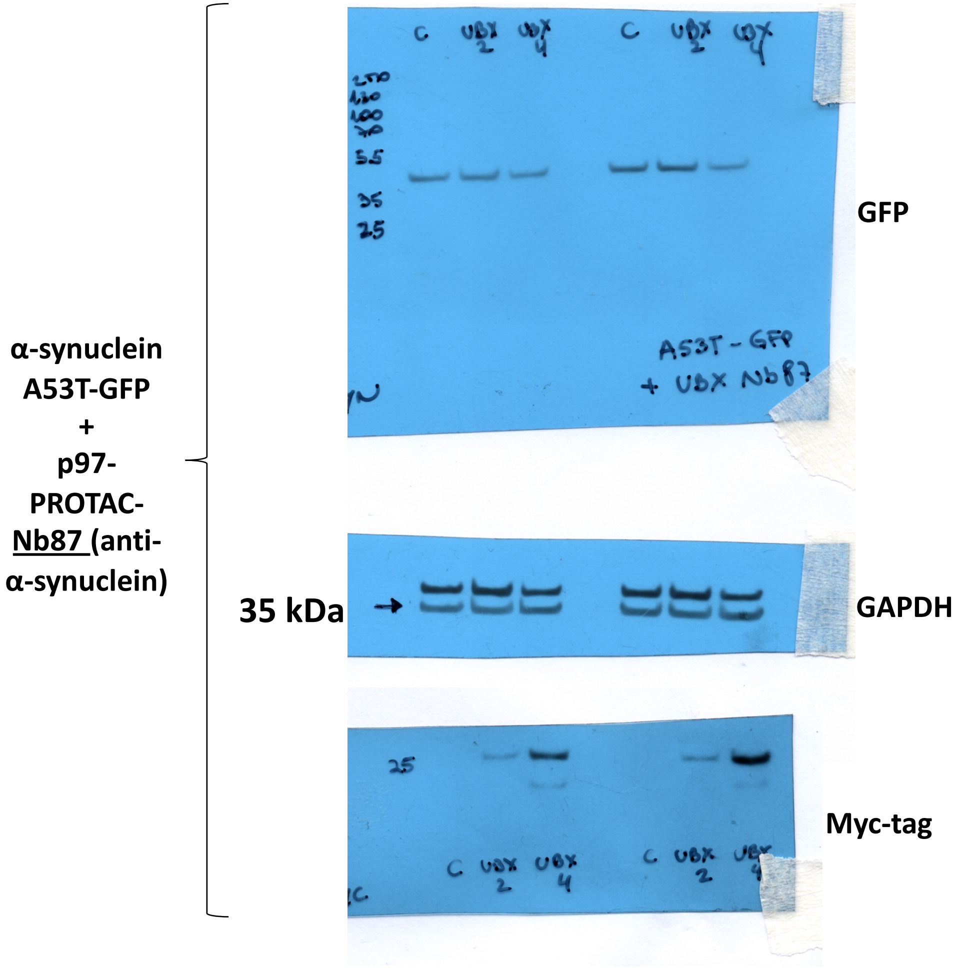

In a parallel experiment, we co-transfected 0.1 µg of **α-synuclein A53T-GFP** with either an **empty vector (C)** or **4 µg of the p97-PROTAC-Nb^{GFP}** construct **(U)**.

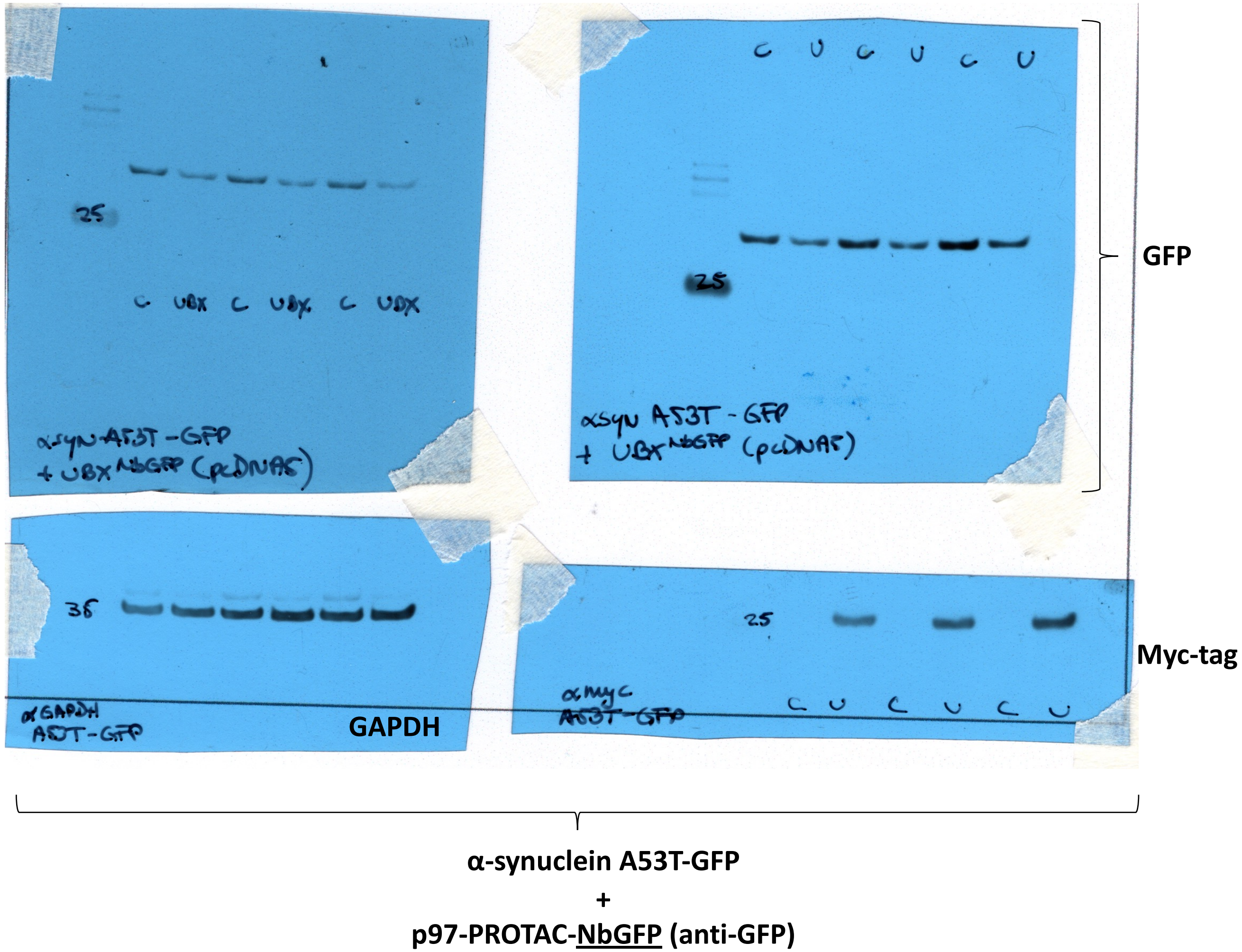

Supplement: Figure 6—source data 2. [file elife-101496-fig6-data2.zip › Figure 6-source data 2/Figure 6A-source data 2.pdf]
