# Supplementary material for: Specific proteolysis mediated by a p97-directed proteolysis-targeting chimera (p97-PROTAC)
Source: eLife. 2025 Nov 26;14:e101496. doi: 10.7554/eLife.101496 (PMC12755880; doi:10.7554/eLife.101496)

Twenty micrograms of protein extracted from cells co-transfected with **untagged α-synuclein A53T** (0.5 μg DNA) and either an **empty vector (C)** or varying amounts of the **p97-PROTAC-Nb87 construct (U)** were loaded. Two independent experiments were performed, each conducted in duplicate using independent samples.

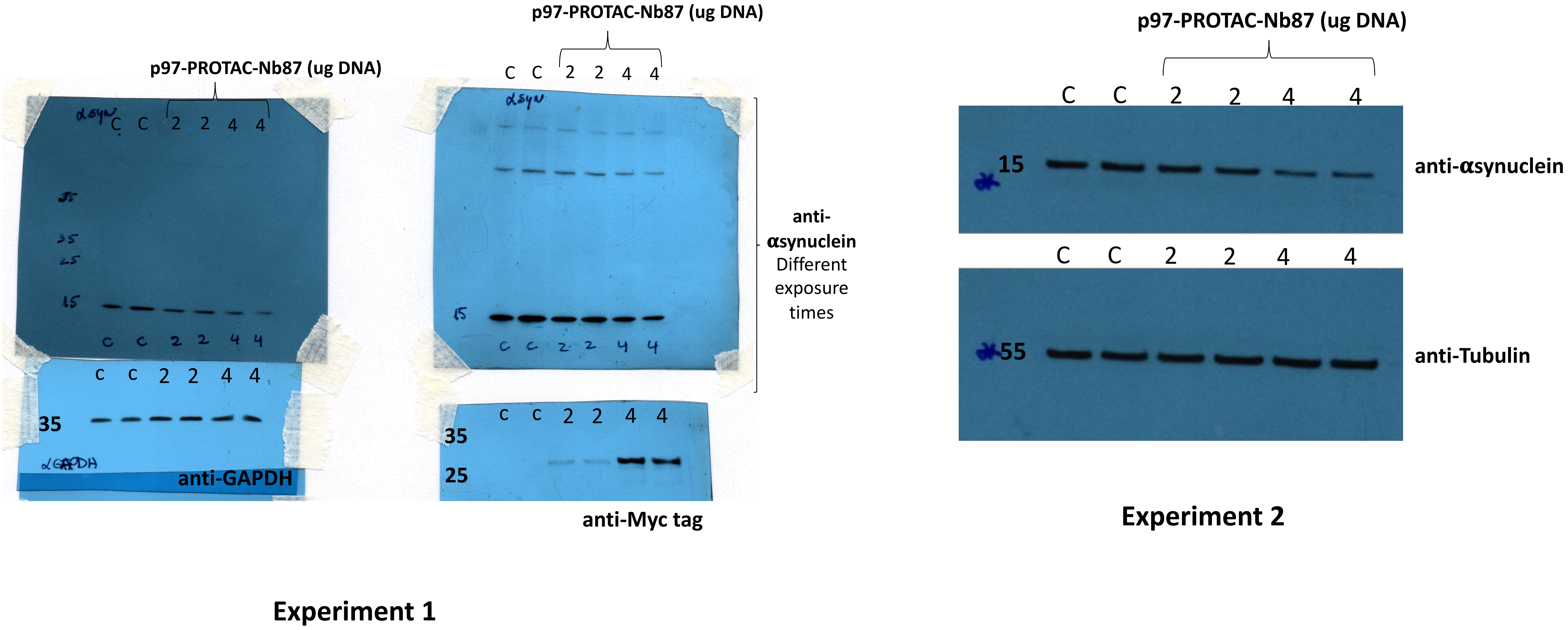

Supplement: Figure 6—source data 2. [file elife-101496-fig6-data2.zip › Figure 6-source data 2/Figure 6C-source data 2.pdf]
